# Why Do People Sometimes Wear an Anonymous Mask? Motivations for Seeking Anonymity Online

**DOI:** 10.1177/01461672231210465

**Published:** 2023-11-24

**Authors:** Lewis Nitschinsk, Stephanie J. Tobin, Deanna Varley, Eric J. Vanman

**Affiliations:** 1 School of Psychology, The University of Queensland, Brisbane, Australia; 2 School of Psychology and Counselling, Queensland University of Technology, Brisbane, Australia

**Keywords:** anonymity, individual differences, motivations, goals, internet/cyberpsychology

## Abstract

Anonymous environments are more accessible than ever. As such, it is important to understand not only how anonymity can change human behavior but also why people are motivated to seek anonymity in online spaces. In four studies, we investigated differences in motivations for seeking anonymity online and their associations with related dispositional factors and online behavior. We found that some people were motivated to seek anonymity to self-express or behave toxically. Both motivations to seek anonymity were associated with low self-concept clarity and high Machiavellianism but differed in their relation to traits such as self-consciousness and psychopathy. Further analyses suggested that people selectively engage in behaviors in anonymous online environments, in line with the specific gratifications they seek through anonymity. We conclude that people seek anonymity to pursue self- or other-related goals that are otherwise more difficult or costly to pursue when identifiable.

“Man is least himself when he talks in his own person. Give him a mask, and he will tell you the truth.”

Wilde, 1891

Psychology has long been fascinated with the veracity of Oscar Wilde’s aphorism. For example, research on anonymity in the last century focused on how being unidentifiable to others affected one’s behavior (Chaum, 1981; Diener, 1979; Festinger et al., 1952). These studies showed that, when people became lost in the crowd and therefore felt anonymous, they were likely to behave differently from when they were not anonymous (Johnson & Downing, 1979; Zimbardo, 1969). A key difference from Wilde’s observation was that anonymous behavior in deindividuation research was not necessarily seen as a person’s “true” behavior but rather the result of conforming to group norms (Reicher, 1987; Spears & Lea, 1994). More importantly, Wilde in 1890, and those researchers in the 20th century, could not have anticipated the internet and how it allows people to interact easily with one another at various levels of anonymity.

Anonymity is often viewed as a continuum, ranging from identifiability to unidentifiability (Christopherson, 2007). Within this continuum are two broad categories: technical anonymity and social anonymity. The former describes complete unidentifiability, whereas the latter describes the perception of anonymity due to a lack of identifiable cues. For example, Milgram (1970) suggested that because city dwellers encounter vast numbers of people daily, the overload of environmental inputs leads to a continual state of heightened anonymity. Similarly, the online world provides many opportunities to be both technically (e.g., commenting on anonymous message boards such as 4chan; Bernstein et al., 2011) and socially (e.g., using a pseudonym on social media platforms such as Reddit) anonymous (van der Nagel & Frith, 2015). As such, many researchers have investigated anonymity’s influence on online behavior (Bargh et al., 2002; Christie & Dill, 2016; Lea & Spears, 1991). Despite this growing body of research, the reasons why people seek anonymity in the first place remain less clear. Identifying such motives would advance our understanding by shifting the focus from how anonymity changes behavior to who is most drawn toward the perceived benefits of anonymous online environments.

## Deindividuation Approaches to Anonymity

Research in the past century demonstrated how anonymous situations could result in deindividuation and changes in human behavior (Festinger et al., 1952; Le Bon, 1896; Zimbardo, 1969). Originally used to explain crowd hooliganism and riots, deindividuation theory broadly aimed to illustrate the fluidity with which humans adhere to societal norms and how such situations often lead to impulsive, aggressive, and antisocial behavior. However, deindividuation does not always have antisocial outcomes; instead, people’s behavior in anonymous environments is influenced by other situational variables such as whether cues for prosocial or antisocial behavior exist (Clark-Gordon et al., 2019; Lea & Spears, 1991; Reicher et al., 1995). In the Social Identity of Deindividuation Effects (SIDE) model, Spears and Lea (1994) proposed that anonymity does not lead to a loss of identity but instead further reinforces and polarizes group norms (Keipi et al., 2016). For example, people tend to conform to group norms in online comments and can more readily morally disengage from instances of cyberbullying when anonymous (Chan et al., 2022; J. E. Chung, 2019). According to the SIDE model, deindividuated situations are not inherently aggressive and can lead to either prosocial or antisocial behaviors. Since its inception, the SIDE model has proved helpful in understanding anonymous online behavior and suggests that one outcome of online anonymity is that people more readily go along with the group’s norms (Hsueh et al., 2015; Perfumi, 2020; Postmes & Spears, 2002).

## A Uses and Gratifications Perspective

Early researchers in the field of cyberpsychology posited that although the online world is socially unique in its anonymity and ability to mitigate the barriers of physical distance, “the internet will always and only be what individuals make of it” (McKenna & Bargh, 2000, p. 72). Indeed, although the internet provides many opportunities to behave anonymously, what people choose to do may be driven by individual differences or dispositional factors (Valkenburg & Peter, 2013). To comprehend the potential motives for seeking anonymity, we first considered its gratifications. Uses and Gratifications Theory (UGT) offers a framework to understand why people seek out certain media to gratify specific wants and needs (Rubin, 2009). Within UGT, both motivations and gratifications are considered key constructs. Motivation refers to the perceived benefits, or what people expect to obtain, from the medium they choose to engage with, whereas gratification refers to what people perceive they have obtained (Rubin, 2009). These gratifications can continue influencing and updating a person’s beliefs or motivations to seek a specific medium, creating a feedback loop (Rayburn & Palmgreen, 1984).

This approach has been beneficial in understanding how online media users control their media consumption, including social media use and podcast listening (Chan-Olmsted & Wang, 2022; Kircaburun et al., 2020). Anonymity is often an affordance of online environments that allow people to interact with media differently. As people actively choose the media they consume, some presumably seek anonymous online environments to reap the perceived benefits of anonymity and use them to pursue self- or other-related goals. Therefore, UGT may be useful when understanding why people seek anonymity online.

## The Dark Side of Anonymity

The internet has long been viewed as a breeding ground for toxicity. For example, people respond more toxically and aggressively in an online chat room when they are anonymous (Lapidot-Lefler & Barak, 2012; Zimmerman & Ybarra, 2016). Although problematic, one perceived benefit of anonymous online environments is the ability to behave antisocially with little fear of reprisal. Certain dark personality traits—Machiavellianism, sadism, and psychopathy—have been popular in explaining the antecedents for various online behaviors. Sadism (the enjoyment of other people’s suffering) and psychopathy (callousness and impulsivity) have both been positively associated with trolling behaviors (Buckels et al., 2014; Craker & March, 2016). Machiavellianism (the callous manipulation of others) has been associated with deceptive online self-presentation (Hart et al., 2019). Those with dark personalities may therefore be motivated to join anonymous online environments to behave toxically or reveal different, more malevolent self-aspects without fear of consequence (Womick et al., 2019).

## Anonymity and the Self

Another gratification of anonymous online environments is the opportunity to self-express and present different, or more “true,” self-versions, much like Oscar Wilde anticipated more than 130 years ago. Indeed, one’s true self-concept is more cognitively accessible while online compared with offline (Bargh et al., 2002). Anonymous online spaces may provide these self-presentational opportunities due to the reduced feelings of vulnerability and judgment from others, along with increased group salience (Grieve & Watkinson, 2016; Spears & Lea, 1994; Turkle, 1995). This may be particularly beneficial for those in marginalized groups as it gives them a place to belong and develop a complete identity (McKenna & Bargh, 1998).

Anonymous environments may also provide additional opportunities for some people to self-disclose. Self-disclosure is vital in maintaining well-being, developing intimacy with others, and increasing likeability (Sprecher et al., 2013). However, self-disclosure carries risks and can potentially have negative ramifications, such as being rejected or outcasted by the listener (Kelly & McKillop, 1996). Therefore, some people—particularly those with low self-esteem—avoid self-disclosing (Leary et al., 1995), leading to long-term stress and reduced well-being. Because anonymous forms of online communication can reduce concern for social evaluation and the threat of negative outcomes, this, in turn, may make it easier for people to self-disclose and reveal self-aspects that they would not reveal while identifiable (Clark-Gordon et al., 2019). For example, people are more likely to spontaneously self-disclose in online environments (Joinson, 2001), an effect that is strengthened when people have a reduced public self-awareness, suggesting that the more anonymous a person feels, the more likely they are to self-disclose spontaneously. Furthermore, those who express their true self over the internet are more likely to create and maintain close relationships, particularly for people with low self-esteem and women low in extraversion who may find online environments a safer and more appealing place to self-disclose (Forest & Wood, 2012; Hamburger & Ben-Artzi, 2000; McKenna et al., 2002).

The gratifications we have described address potential motives for anonymously interacting with others. These gratifications are centered around how anonymity may make it easier for people to engage with others in specific ways. Of course, anonymity may also afford practical gratifications. For instance, some individuals browse websites and download files anonymously due to privacy concerns or to avoid personalized advertising (Kang et al., 2013). Others conceal their identity to maintain a professional public image (Wang et al., 2018). Such gratifications are focused more on users’ technical anonymity. The more anonymous they are, the more likely people can prevent spam offers, protect their bank accounts, avoid companies sharing their information, and so on. In this article, we are specifically interested in gratifications that underlie a person choosing to engage in social interactions while anonymous. Therefore, this article’s scope is on motivations centered around these social interactions.

## Overview

Research has sporadically assessed motivations to seek anonymity. However, fewer studies have attempted to compare these motivations directly and have done so through qualitative interviews or single-item measures (Kang et al., 2013; Keipi et al., 2015). Most research on anonymity has instead focused on situational and contextual factors, specifically aiming to understand how anonymity changes behavior. Although situational and contextual factors are theoretically crucial to our understanding of anonymous behavior, online environments have made anonymity more accessible than ever. It is important to understand what leads people to seek anonymity. Identifying why people are motivated to seek anonymity advances our knowledge about who engages in anonymous online activity and provides an important context for understanding how people behave when they are anonymous. Therefore, this research aimed to investigate why people seek anonymity in online spaces, identify related personality traits and dispositional factors to shed further light on these motives, and examine how these motives predict self-reported anonymous online behavior.

We tested these predictions across four studies. In Studies 1 and 2, we developed and refined a self-report questionnaire examining motivations for seeking anonymity when interacting with others. We report Study 3 (preregistered) in three parts. Study 3a validated the scale through confirmatory factor analysis. Study 3b assessed the new subscales’ convergent validity and explored theoretically relevant individual differences. Study 3c assessed how anonymous motivations predict self-reported anonymous online behavior and time spent online. Finally, in Study 4 (preregistered), we ran a 7-day diary study to examine the relationships between anonymous motivations and self-reported online behavior. Statistical analyses for all studies were conducted using R version 4.0.3 (R Core Team, 2017). We report how we determined our sample size, all data exclusions (if any), and all measures used for each study. All studies were approved by the institutional ethics review board. Readers can access the preregistration for Study 3 (https://osf.io/5fjxw/?view_only=613a57c25ca34996a9ce9a744e42279a) and Study 4 (https://osf.io/srh2d/?view_only=a859c71748694489991e427a7f64b172). Supplemental materials for all studies, including data sets, codebooks, items, and stimuli, are accessible at https://osf.io/gqpbc/?view_only=09297f31eb074c619cf2541ccac9d4e8.

## Studies 1 and 2: Development of the Online Anonymity Questionnaire

In Studies 1 and 2, we aimed to develop a measure assessing motivations to seek anonymity. Items were created and refined through extensive discussions and brainstorming sessions among three authors familiar with the anonymity and cyberpsychology literature. Potential items were generated by reviewing past research on the perceived benefits of anonymous online behavior (Christie & Dill, 2016; Christopherson, 2007; Craker & March, 2016; Dolev-Cohen, & Barak, 2013; Joinson, 2001; Lapidot-Lefler & Barak, 2012; Misoch, 2015; Rains & Keating, 2015). Items assessed motivations by asking participants about the perceived benefits of anonymity and the behaviors people prefer to enact when anonymous. Assessing the perceived benefits of anonymity directly addresses the gratifications a person may—or may not—seek in anonymous environments. Likewise, assessing behavior directly addresses the perceived gratifications obtained, which, in turn, can influence a person’s beliefs or attitudes toward anonymous environments.

### Method

#### Participants

In Study 1, 242 university undergraduates participated for course credit. Fifteen participants were excluded from the sample as they did not complete the entire questionnaire meaning the final sample included 227 participants (160 women; 67 men, *M_age_* = 21.26, *SD* = 3.79). This sample size surpassed the minimum subject-to-item ratio of 5:1 (Kyriazos, 2018). In Study 2, 416 (237 women; 175 men; 4 undisclosed; *M_age_* = 33.55, *SD* = 11.95) Prolific participants living in the United Kingdom, the USA, Canada, or Australia participated in exchange for payment. The sample size surpassed a more stringent subject-to-item ratio of 10:1 (Boateng et al., 2018; Clark & Watson, 1996). In both studies, all participants were fluent in English.^1^

#### Measure and Procedure

In Study 1, the initial item pool for the Online Anonymity Questionnaire (OAQ) included 30 items assessing anonymous motivations (e.g., Being anonymous allows me to experiment with new ideas). In Study 2, we further developed and refined items within the scale. The OAQ in Study 2 included 19 items.^2^ In both studies, the OAQ was rated on a five-point scale (1 = *strongly disagree*, 5 = *strongly agree*). The OAQ was presented to participants in an online questionnaire format. The order of the items was randomized for each participant. The instructions for the OAQ specified that the questionnaire was focused on why people are sometimes anonymous on the internet.

#### Statistical Approach

For Study 1 and Study 2, an exploratory factor analysis (EFA) was conducted with Promax rotation and principal axis factoring. The scale’s factor solution was chosen using parallel analysis. No items showed high intercorrelations (*r* > .|90|) or excessive skew (>|2|). Items with target loadings <.|30,| or cross-loadings >.|30| were removed (Carpenter, 2018; Clark & Watson, 2019; Costello & Osborne, 2005).

### Results

#### Study 1

A six-factor solution was initially revealed; however, a three-factor solution was retained as it was more theoretically parsimonious, and few items were loaded outside these factors. The EFA retained 25 items and explained 48% of the variance. Assumptions of sampling adequacy (Kaiser–Myer–Olkin = .90) and sphericity were met χ^2^(435) = 3,321.02, *p* < .001. Four items were removed for not loading significantly onto any factor, and one was removed due to high cross-loadings. See Table A for the supplemental information for factor loadings. The first factor (14 items) reflected seeking anonymity for self-expression motives, including self-disclosure and self-presentation strategies (e.g., “I feel like I can be someone else when I’m anonymous”). The second factor (8 items) reflected online toxicity (e.g., “When I’m anonymous I do things that are normally unacceptable in society”). Finally, the third factor (three items) reflected a lower preference for anonymity; however, this factor was removed as it was not defined by clear factor loadings and showed low internal reliability (α = .44).

Means, standard deviations, correlations between subscales, and Cronbach’s coefficient alphas are reported in Table 1. The two subscales assessing motivations for anonymity were positively correlated. However, we believe that some items could be improved to better represent the nature and structure of their respective construct (Clark & Watson, 2019). As a result, we decided to rewrite several items and run a secondary EFA with a new sample. Specifically, four new items were created, three items were rewritten, and seven items were deleted due to vague and inaccurate expressions. Please refer to the supplemental information for a complete list of initial items and a full explanation of changes between Study 1 and 2.

**Table 1.** Means, Standard Deviations, Cronbach’s Alphas, and Correlations Between Factors of the Online Anonymity Questionnaire (OAQ) in Studies 1 to 4.

	Anonymous self-expression	Anonymous toxicity
Study 1 (*N* = 227)		
Anonymous Toxicity	.50***	
*M*	3.42	2.14
*SD*	0.90	0.79
Cronbach’s Alpha (α)	.93	.82
Study 2 (*N* = 416)		
Anonymous Toxicity	.42***	
*M*	3.48	2.01
*SD*	0.83	0.91
Cronbach’s Alpha (α)	.91	.87
Study 3 (*N* = 321)		
Anonymous Toxicity	.56***	
*M*	3.18	2.11
*SD*	0.96	0.87
Cronbach’s Alpha (α)	.92	.84
Study 4 (*N* = 325)		
Anonymous Toxicity	.49***	
*M*	3.07	2.10
*SD*	0.76	0.71
Cronbach’s alpha (α)	.86	.77

*** *p* < .001.

#### Study 2

A two-factor solution was retained, including 16 items explaining 52% of the variance. Assumptions of sampling adequacy (Kaiser–Myer–Olkin = .90) and sphericity were met χ^2^(325) = 3659.19, *p* < .001. See Table 2 for factor loadings. One item was removed due to low factor loadings, and one item was removed due to low communalities (< .40; Costello & Osborne, 2005). In addition, we removed the item “I think being anonymous could allow me to be famous.” Best practices in scale development rely on both statistical decision-making and theoretical logic (Carpenter, 2018). As a result, this item was removed because we believed its wording was more closely related to motivations for notoriety rather than motivations to be toxic.

**Table 2.** Factor Loadings of the Exploratory Factor Analysis (Study 2).

Number	Item	Anonymous self-expression	Anonymous toxicity
SE1	I feel more comfortable disclosing information about my ideas, thoughts and feelings when I am anonymous.	**.705**	−.173
SE2	Being anonymous allows me to share thoughts and feelings I otherwise wouldn’t share with people who know me.	**.798**	−.170
SE3	Being anonymous allows me to experiment with new ideas.	**.720**	−.068
SE4	I feel like I can be somebody else when I’m anonymous.	**.665**	.082
SE5	I can present myself in a different way when I’m anonymous.	**.755**	.051
SE6	Being anonymous online allows me to escape or distract myself from reality.	**.650**	.134
SE7	When I am anonymous online, I can talk to people who wouldn’t normally talk to me in the offline world.	**.623**	.119
SE8	Being anonymous allows me to join groups I wouldn’t normally join in the real world.	**.663**	.054
SE9	I can connect with people I normally wouldn’t when I’m anonymous.	**.777**	.032
SE10	I feel like I can express my true self when I’m anonymous.	**.693**	.050
TOX1	I am more likely to do things that are unlawful or illegal when I am anonymous.	−.042	**.730**
TOX2	When I’m anonymous I do things that are normally unacceptable in society.	−.034	**.749**
TOX3	I get satisfaction from aggravating people anonymously online.	−.098	**.761**
TOX4	Being anonymous online is fun because I don’t get in trouble for what I say.	.080	**.721**
TOX5	When I am anonymous, I find it easier to trick and manipulate people.	.012	**.713**
TOX6	I like being anonymous because I can say whatever I want without consequences.	.049	**.711**

*Note.* Factor loadings reflect values from the pattern matrix. Bolded coefficients denote primary factor loadings. *SE* = Anonymous Self-Expression, TOX = Toxic Anonymity.

The themes of each factor were consistent with Study 1. Factor 1 (10 items) reflected anonymous self-expression motives. Factor 2 (6 items) reflected motives to behave toxically while anonymous. Both subscales demonstrated good internal consistency (α > .8) and were positively correlated (*r* = .42, *p* < .001).

### Discussion

Studies 1 and 2 found that people were motivated to seek anonymity for different outcomes or perceived gratifications. First, some people were motivated to be anonymous to present or experiment with self-aspects that they would not feel comfortable expressing while identifiable. Second, some people were motivated to be anonymous to behave toxically and reveal more malevolent self-aspects. Interestingly, these two factors were positively correlated, indicating potential similarities in people seeking anonymity online generally, even if the motivations themselves are distinct. Importantly, Study 2 saw both factors remain internally consistent.

## Study 3

Study 3 had three aims. First, we aimed to confirm the reliability of the OAQ through a confirmatory factor analysis (Study 3a). Second, we aimed to assess the convergent and discriminant validity of the OAQ and explore each subscale’s relationship with theoretically related individual differences (Study 3b). Third, we aimed to test the predictive validity of the OAQ by assessing the relationship between its subscales and self-reported anonymous online behavior (Study 3c). The same participants completed Studies 3a, 3b, and 3c in a single session. On average, participants took 26 min to complete the survey.

### Participants

A sample of 322 Prolific participants (123 women; 191 men; 5 non-binary; 3 undisclosed) aged between 18 and 71 (*M* = 33.00, *SD =* 11.69), and living in the United Kingdom, the United States, Canada, or Australia participated in exchange for payment. All participants were fluent in English. The sample size was determined by a minimum of 10 participants per item (Boateng et al., 2018; Clark & Watson, 1996). One participant was excluded for failing to answer all items on the OAQ (final *N* = 321). In the sample, 65% identified as White, 15% Asian, 7% African American, 5% Hispanic/Latino, and 1% Other.

### Procedure

After providing informed consent, participants read that the purpose of the study was to investigate why people are sometimes anonymous on the internet. After completing a series of demographic questions, participants first completed the 16-item OAQ. Participants also completed additional self-report measures assessing personality characteristics and their online behavior. These will be reported in Study 3b and Study 3c.

## Study 3a: Validation of the OAQ

Study 3a aimed to confirm the consistency and stability of the factor structure established in Study 2. As such, a confirmatory factor analysis was conducted.

### Results

**Figure 1:**
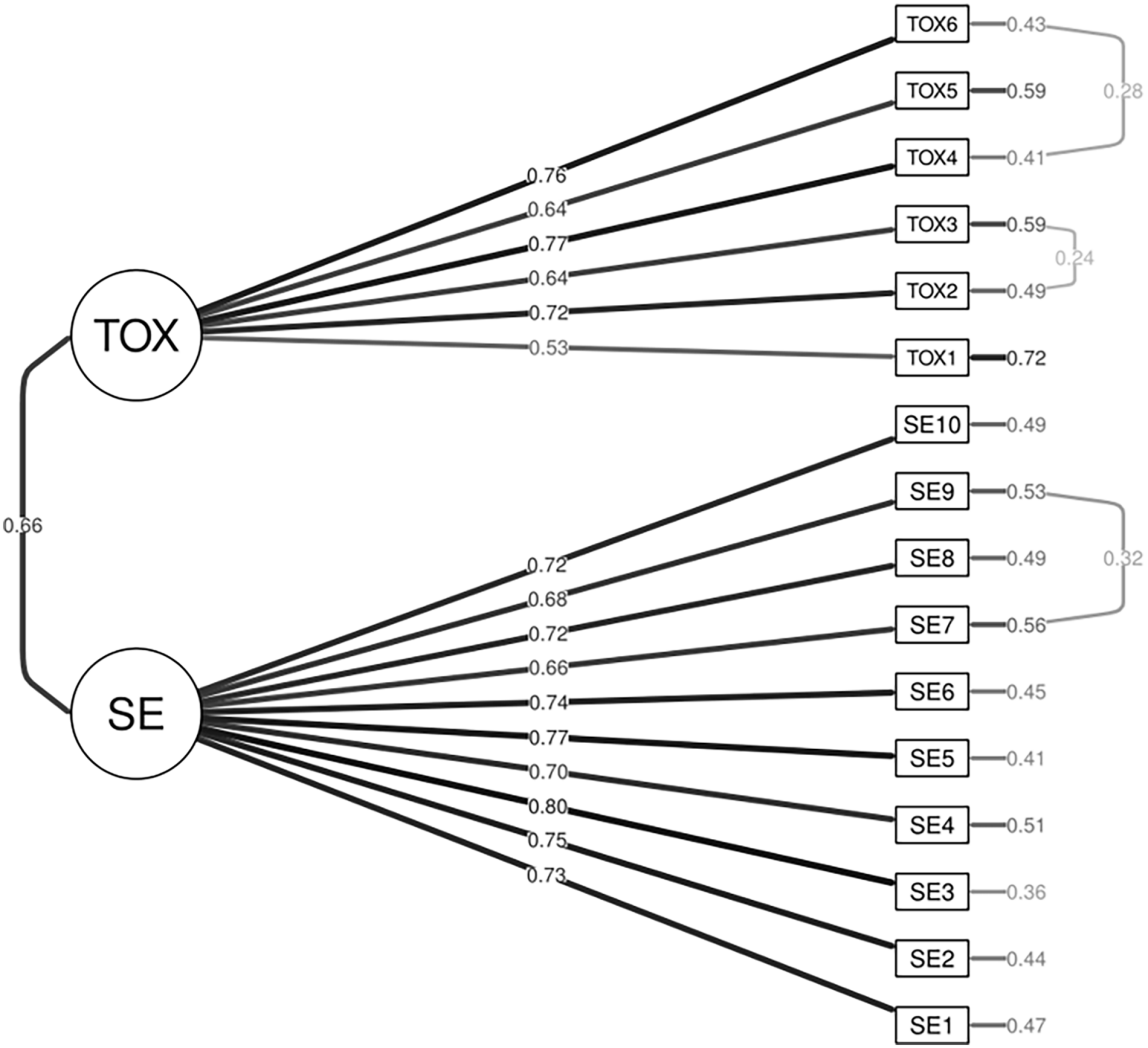
Path Diagram for the Two-Factor OAQ in Study 3a *Note.* Parameter constraints were added between three item pairs to improve model fit due to larger-than-expected covariances. OAQ = Online Anonymity Questionnaire; TOX = Toxic Anonymity; *SE* = Anonymous Self-Expression.

The lavaan package was used to conduct a confirmatory factor analysis (Rosseel, 2012). To evaluate model fit, the comparative fit index (CFI), standardized root mean residual (SRMR), and the root mean squared error of approximation (RMSEA) were used. To determine an acceptable fit, we used the following cut-offs: CFI > 0.90, RMSEA < 0.07, and SRMR < 0.08 (Cheung & Rensvold, 2002). First, all items were entered as loading on a single factor. The overall model fit for a one-factor solution was poor: χ^2^(104) = 655.95, *p* < .001, CFI = .792, RMSEA = .129, SRMR = .03. Next, we aimed to fit the two-factor structure identified in Study 2. The two-factor solution significantly improved model fit χ^2^(103) = 318.38, *p* < .001, CFI = .919, RMSEA = .081, SRMR = .057. However, several fit indices showed larger-than-expected covariances not captured by the initial model structure. As such, modification indices were examined to assess misfit. Modification indices suggested that three item pairs showed larger than expected covariances, potentially due to the similarity in the items’ wording or content. Parameter constraints were freed for these three item pairs (see Figure 1).

The modified two-factor structure demonstrated improved model fit, χ^2^(100) = 258.43, *p* < .001, χ^2^Δ (100) = 59.95, *p* < .001, CFI = .940, RMSEA = .070, SRMR = .053. Loadings for the anonymous self-expression subscale ranged between .66 and .80, and between .53 and .77 for the toxic anonymity subscale (see Table 3). Both self-expression and toxic anonymity motives were again positively correlated. Means, standard deviations, intercorrelations, and alphas showed few differences between the EFA and the CFA samples (see Table 1). For item-level correlations, please see Table B of the supplemental information.

**Table 3.** Descriptive Statistics of Observed Variables and Factor Loadings (Study 3a).

Number	Items	*M*	*SD*	Loadings
SE1	I feel more comfortable disclosing information about my ideas, thoughts, and feelings when I am anonymous.	3.37	1.30	.727
SE2	Being anonymous allows me to share thoughts and feelings I otherwise would not share with people who know me.	3.23	1.31	.751
SE3	Being anonymous allows me to experiment with new ideas.	3.24	1.26	.798
SE4	I feel like I can be somebody else when I am anonymous.	2.88	1.27	.700
SE5	I can present myself in a different way when I’m anonymous.	3.28	1.22	.766
SE6	Being anonymous online allows me to escape or distracted myself from reality.	3.02	1.35	.744
SE7	When I am anonymous online, I can talk to people who wouldn’t normally talk to me in the offline world.	3.27	1.28	.662
SE8	Being anonymous allows me to join groups I wouldn’t normally join in the real world.	3.08	1.31	.715
SE9	I can connect with people I normally wouldn’t when I’m anonymous.	3.37	1.23	.683
SE10	I feel like I can express my true self when I am anonymous.	3.09	1.24	.719
TOX1	I am more likely to do things that are unlawful or illegal when I am anonymous.	1.96	1.18	.528
TOX2	When I’m anonymous I do things that are normally unacceptable in society.	1.91	1.13	.715
TOX3	I get satisfaction from aggravating people anonymously online.	1.69	1.05	.637
TOX4	Being anonymous online is fun because I don’t get in trouble for what I say.	2.40	1.22	.765
TOX5	When I am anonymous, I find it easier to trick and manipulate people.	2.30	1.19	.644
TOX6	I like being anonymous because I can say whatever I want without consequences.	2.39	1.26	.757

*Note. SE* = Anonymous Self-Expression; TOX = Toxic Anonymity.

Independent *t* tests were conducted to investigate gender differences within each subscale (see Table 4). Men scored higher on anonymous toxicity than women (*p* < .001). No differences were found for anonymous self-expression (*p* = .139). Anonymous self-expression (*r* = −.28, *p* < .001) and toxic anonymity (*r* = −.16, *p* = .006) were negatively associated with age. Finally, tests of configural, metric, and scalar invariance showed psychometric equivalence of the OAQ across both gender and age (see Table 5).

**Table 4.** Gender Differences Across the Online Anonymity Questionnaire (Study 3a).

Type	Anonymous self-expression	Anonymous toxicity
Male	3.25 (0.93)^a^	2.28 (0.91)^a^
Female	3.08 (1.01)^a^	1.85 (0.76)^b^

*Note.* Variations in superscripts within columns indicate that means are significantly different.

**Table 5.** Tests of Invariance for Age and Gender Across the Online Anonymity Questionnaire in Study 3a.

Type	Model	*df*	χ^2^	CFI	RMSEA	Δ χ^2^	ΔCFI	ΔRMSEA
Gender	Configural	200	286.33	.950	.053			
	Metric	214	280.55	.962	.045	−5.78^a^	.012	−.008
	Scalar	228	297.37	.960	.044	16.82^a^	.002	−.001
Age	Configural	200	307.56	.940	.058			
	Metric	214	272.13	.968	.041	−35.43^b^	.028	−.017
	Scalar	228	285.45	.968	.040	13.32^b^	.000	−.001

*Note.* Changes in the comparative fit index (CFI) and root mean square error of approximation (RMSEA) are relative to the previous model (i.e., metric vs. configural; scalar vs. metric). Variations in superscripts within columns indicate that means are significantly different. CFI = comparative fit index; RMSEA = root mean square error of approximation.

### Test–Retest Reliability

An additional study (*N* = 168, pre-registered) was conducted to assess the test–retest reliability of the OAQ across a four-week interval. Anonymous self-expression motives (*r* = .75, *p* < .001) and anonymous toxicity motives (*r* = .79, p < .001) showed acceptable test–retest reliability. See Supplemental Information for participant information.

### Discussion

Overall, the psychometric properties for the 16-item OAQ—developed across three studies with 965 participants—indicate strong model fit and internal reliability. Two distinct subscales were identified: anonymous self-expression and toxic anonymity. The results suggest different motivations to seek anonymity rather than a unidimensional factor reflecting anonymous motivations. Self-expression and toxic anonymity motives were positively correlated, suggesting that similarities exist for people seeking anonymity, even if their motives differ. Establishing a reliable measure to assess motivations to seek anonymity online allows us to investigate its convergent and discriminant validity and explore its relationship with theoretically relevant constructs.^3^

## Study 3b: Anonymous Motivations and Individual Differences

Study 3b aimed to assess the OAQ’s convergent and discriminant validity and explore theoretically relevant constructs in why people seek anonymity online. First, we evaluated the OAQ’s relationship with online self-presentation styles and beliefs using the Presentations of Online Self Scale for Adults (POSSA; Fullwood et al., 2016; Strimbu et al., 2021). As affordances of online spaces (e.g., anonymity) allow people to experiment with their self-presentation (Nitschinsk et al., 2022a), people may be motivated to seek anonymity to present self-aspects that they cannot present while identifiable. We predicted that anonymous self-expression and toxicity motives would correlate with the presentation of an adaptable, inauthentic self and beliefs that online environments benefit self-presentation.

Next, we assessed the relationships between the OAQ and several self-related individual differences. These include social anxiety, self-esteem, self-concept clarity, acquisitive and protective self-monitoring, and public and private self-consciousness. Previous research has suggested that socially anxious people with a negative or uncertain self-view may be attracted to anonymity as it allows them to self-disclose or experiment without fear of social evaluation (Bargh et al., 2002; Forest & Wood, 2012; Khan et al., 2016; Weidman et al., 2012). Therefore, we predicted that those high in protective self-monitoring or social anxiety, or low in self-esteem or self-concept clarity, may be more motivated to seek anonymity online, particularly to self-express. Anonymous environments have been shown to heighten private self-awareness, reduce public self-awareness, and increase self-disclosure (Hirsh et al., 2011; Joinson, 2001). Therefore, at a trait level, we predicted those higher in public and private self-consciousness might be more motivated to seek anonymity to self-express.

Another set of predictions was centered around dark personality traits. Machiavellianism has been associated with utilizing deceptive identities and strategic or exaggerated forms of self-presentation in online environments (Bolino & Turnley, 2003; Drouin et al., 2016; Hart et al., 2019). Therefore, we predicted that Machiavellianism would be related to anonymous toxicity and anonymous self-expression. Further, trait psychopathy and sadism have been consistently associated with antisocial online behaviors (Buckels et al., 2014; Craker & March, 2016). Therefore, we predicted that people higher in these traits would be more motivated to behave toxically while anonymous online.^4^

Finally, we assessed the relationship between the OAQ and personality using the HEXACO (Ashton & Lee, 2007). The HEXACO assesses honesty-humility, emotionality, extraversion, agreeableness, conscientiousness, and openness to experience. Various personality dimensions have been associated with differences in online behavior; therefore, we predicted these differences might extend to anonymous motivations.

Honesty-humility has been argued to be an umbrella term for dark-personality traits (Jones & Paulhus, 2017). As such, we expected honesty-humility to be negatively associated with anonymous self-expression and toxicity motives, as both motives involve potentially deceitful presentation strategies. We predicted this negative relationship would be stronger for toxic motives, as the toxic motives are more deviant and insincere than the self-expression motives. We also predicted that agreeableness would be negatively associated with anonymous toxicity motives, as previous research has found cyberbullying and online trolling to be associated with low agreeableness (McCreery & Krach, 2018; Zezulka & Seigfried-Spellar, 2016).

We predicted that extraversion would be negatively associated with anonymous self-expression, as people who are low in extraversion are more likely to explore their self-presentation online (Michikyan et al., 2014). Finally, as emotionality is partly associated with anxiousness (Ashton & Lee, 2007), we predicted that the trait would be positively related to anonymous self-expression motives. We did not have a priori predictions for conscientiousness or openness to experience in relation to the OAQ.

### Measures

For scale reliabilities and descriptive statistics in Study 3b, see Table 6. To assess online presentation styles and beliefs, we used the POSSA (Fullwood et al., 2016; Strimbu et al., 2021). The scale consists of 17 items measured on a five-point scale (1 = *strongly disagree*, 5 = *strongly agree*) and is composed of three subscales: Adaptable self, authentic self, and freedom of self.

**Table 6.** Scale Reliabilities (Cronbach’s Alpha) and Descriptive Statistics in Study 3 and Study 4.

Scale	*M*	*SD*	Study 3		*M*	*SD*	Study 4	
			Range	α			Range	α
Self-Concept Clarity	3.23	0.97	1.00–5.00	.92	2.77	0.67	1.08–4.86	.83
Self-Esteem	2.72	0.74	1.00–4.00	.94	2.70	0.53	1.10–4.00	.89
Dark Triad								
Narcissism	2.50	0.66	1.00–4.22	.73	2.69	0.60	1.33–4.56	.73
Machiavellianism	3.01	0.73	1.33–5.00	.80	2.90	0.63	1.33–4.44	.76
Psychopathy	2.10	0.68	1.00–5.00	.77	2.02	0.58	1.00–3.89	.73
Sadism	1.13	0.18	1.00–1.90	.78	1.90	0.12	1.10–2.00	.70
Self-Monitoring								
Acquisitive	2.60	0.81	1.00–5.00	.73				
Protective	3.00	0.73	1.00–5.00	.76				
Self-Consciousness								
Private	1.82	0.52	0.00–3.00	.72				
Public	1.79	0.70	0.00–3.00	.86				
Social Anxiety	1.77	0.83	0.00–3.00	.86				
HEXACO								
Honesty/Humility	3.47	0.73	1.00–5.00	.76				
Emotionality	3.28	0.69	1.30–5.00	.76				
Extraversion	2.83	0.82	1.00–4.90	.85				
Agreeableness	3.23	0.72	1.10–4.90	.82				
Conscientiousness	3.60	0.69	1.80–5.00	.82				
Openness	3.71	0.69	1.80–5.00	.78				
Online Self-Presentation								
Freedom of Self	3.04	0.85	1.00–5.00	.62				
Authentic Self	3.67	0.78	1.50–5.00	.58				
Adaptable Self	2.28	1.01	1.00–5.00	.87				

*Note.* Study 3 *N* = 321, Study 4 *N* = 325. α = Cronbach’s alpha.

We also assessed several self-related variables. Acquisitive and protective self-monitoring were assessed using the revised 13-item self-monitoring scale (Snyder, 1974; Wilmot et al., 2017). Self-concept clarity was assessed using the 12-item self-concept clarity scale (Campbell et al., 1996). Self-monitoring and self-concept clarity were measured on a 5-point scale (1 = *strongly disagree*, 5 = *strongly agree).* Self-esteem was assessed using the 10-item Rosenberg self-esteem scale and was measured on a 4-point scale (1 = *strongly disagree*, 4 = *strongly agree*; Rosenberg, 1965). Public self-consciousness, private self-consciousness, and social anxiety were assessed using the 22-item revised self-consciousness scale and were measured on a 4-point scale (0 = *not like me at all, 3 = a lot like me*; Scheier & Carver, 1985).

We assessed narcissism, Machiavellianism, and psychopathy using the Short Dark Triad (SDT) scale (Jones & Paulhus, 2014). The SDT is a 27-item scale. Items were assessed on a five-point scale (1 = *strongly disagree*, 5 = *strongly agree)*. We assessed sadism using the 10-item Short Sadistic Impulse Scale, measured on a two-point scale (0 = *disagree*, 1 = *agree;* O’Meara et al., 2011). Finally, personality was assessed using the 60-item HEXACO, measured on a 5-point scale (1 = *strongly disagree*, 5 = *strongly agree)*. It assesses six personality traits: honesty/humility, emotionality, extraversion, agreeableness, conscientiousness, and openness to experience (Ashton & Lee, 2007).

### Data Analysis

The correlations between the OAQ subscales and all other variables are presented in Table 7. A Benjamini-Hochberg correction was made to reduce the possibility of Type 1 Error. *Z-*score differences between anonymous self-expression and anonymous toxicity correlations were correlated using the “cocor” package (Diedenhofen & Musch, 2015). Regression analyses were conducted with narcissism, Machiavellianism, psychopathy, and sadism as predictors and each OAQ subscale as outcome variables. We used semi-partial correlations to determine the effect size of each predictor.

**Table 7:** Correlations Between Motives to Seek Anonymity, Individual Differences, and Online Behavior in Study 3 and Study 4.

	Study 3			Study 4		
	Self-expression	Toxicity	Difference (*Z*)	Self-expression	Toxicity	Difference (*Z*)
Self-Concept Clarity	**−.42**	**−.33**	−1.89	**−.39**	**−.18**	**−3.98**
Self-Esteem	**−.31**	**−.23**	−1.72	**−.29**	−.03	**−4.62**
Dark Triad						
Narcissism	−.11	.10	**−3.90**	.01	**.20**	**−3.53**
Machiavellianism	**.47**	**.47**	−0.12	**.41**	**.41**	−0.12
Psychopathy	**.25**	**.56**	**−6.64**	**.23**	**.50**	**−5.24**
Sadism	**.20**	**.37**	**−3.33**	**.26**	**.31**	0.86
Self-Monitoring						
Acquisitive	**−.12**	−.02	**−1.98**			
Protective	**.46**	**.31**	**3.01**			
Self-Consciousness						
Private	**.17**	−.01	**3.66**			
Public	**.23**	.06	**3.27**			
Social Anxiety	**.36**	**.18**	**3.60**			
HEXACO						
Honesty/Humility	**−.28**	**−.44**	**3.29**			
Emotionality	.03	**−.18**	**4.23**			
Extraversion	**−.35**	**−.15**	**−4.03**			
Agreeableness	**−.14**	**−.25**	**2.17**			
Conscientiousness	**−.26**	**−.36**	**2.13**			
Openness	**−.01**	**−.25**	**4.80**			
Online Self-Presentation						
Freedom of Self	**.72**	**.39**	**8.06**			
Authentic Self	**−.40**	**−.46**	1.27			
Adaptable Self	**.61**	**.62**	−0.07			
Anonymous Online Behavior						
Benign Behavior	**.47**	**.33**	**2.90**	**.24**	**.14**	1.08
Malign Behavior	**.37**	**.58**	**−4.78**	.10	**.20**	−1.61
Identifiable Online Behavior						
Benign Behavior				−.09	**−.15**	1.81
Malign Behavior				−.09	**.18**	−1.84

*Note.* Study 3 *N* = 321, Study 4 *N* = 325, correlations were corrected using the Benjamini-Hochberg procedure to control type 1 error. Reported p values have been adjusted so that *p* < .05 is significant given the number of tests performed. Bolded correlations and *Z*-score differences are significant (*p < .05*). For Study 3, Pearson’s *r* values outside the range of ±0.11 are *p* < .05, ±0.14 are *p* < .01, and ±0.18 are *p* < .001. For Study 4, Pearson’s *r* values outside the range of ±0.11 are *p* < .05, ±0.14 are *p* < .01, and ±0.17 are *p* < .001.

### Results

#### Presentation of Online Self

As hypothesized, anonymous self-expression and anonymous toxicity were positively associated with freedom of self-presentation and adaptable self-presentation and negatively related to authentic self-presentation. The correlation strength for freedom of self-presentation was significantly stronger for anonymous self-expression. Adaptable and authentic self-presentations showed no significant differences in their correlation strengths.

#### Self-Related Individual Differences

As predicted, anonymous self-expression was positively correlated with protective self-monitoring, private self-awareness, public self-awareness, and social anxiety. It was negatively correlated with acquisitive self-monitoring, self-concept clarity, and self-esteem. Toxic anonymity was positively correlated with protective self-monitoring and social anxiety and negatively correlated with self-concept clarity and self-esteem. Self-esteem and self-concept clarity showed no significant differences in their correlation strengths, all other correlations were significantly stronger for anonymous self-expression.

#### Other Personality Variables

Anonymous self-expression and anonymous toxicity were positively correlated with Machiavellianism, psychopathy, and sadism. All Dark Tetrad Traits, except Machiavellianism, showed significant differences in their correlation strengths. To control for shared variance between the dark personality traits, we conducted a series of regression analyses to examine the dark tetrad traits as predictors of each OAQ subscale. Machiavellianism positively predicted anonymous self-expression with a medium effect size, whereas narcissism negatively predicted anonymous self-expression with a small effect size. Toxic anonymity motives were positively associated with both Machiavellianism and psychopathy, with small and large effect sizes, respectively (see Table 8).

**Table 8.** Multiple Regression Analysis Between Dark Tetrad Traits and the Online Anonymity Questionnaire (OAQ) (Study 3b).

Variable	Anonymous self-expression			Toxic anonymity		
	*b*	*SE*	*r* [95% CI]	*b*	*SE*	*r* [95% CI]
Intercept	1.57	.34		−.05	.29	
Narcissism	−.30***	.08	−0.22 [−0.32, –0.11]	−.13	.06	−0.11 [−0.22, –0.00]
Machiavellianism	.63***	.08	0.41 [0.32, 0.50]	.27***	.07	0.23 [0.12, 0.33]
Psychopathy	.02	.10	0.01 [−0.10, 0.12]	.54***	.08	0.35 [−0.25, 0.44]
Sadism	.38	.31	0.07 [−0.04, 0.18]	.47	.25	0.10 [−0.01, 0.21]
	*F*(4, 316) = 28.3***			*F*(4, 316) = 45.6***		
	*R*^2^ =.264			*R*^2^ =.366		

*Note. N* = 321. Each column represents a separate regression with variables in rows as predictor variables, and variables in the column header as outcome variables. CI = confidence interval.

*** *p* < .001.

When assessing HEXACO personality traits, anonymous self-expression was negatively associated with honesty-humility, agreeableness, conscientiousness, and extraversion. Toxic anonymity was negatively associated with honesty-humility, emotionality, extraversion, agreeableness, conscientiousness, and openness to experience. The correlation strength for extraversion was significantly stronger for anonymous self-expression, all other correlation strengths were significantly stronger for anonymous toxicity.

### Discussion

#### The Self

As expected, those motivated to seek anonymity—either for self-expression or toxic motives—were more likely to present adaptable or inauthentic self-versions while online and believed that online environments afforded them the freedom to self-present differently. In addition, these motives were associated with having a more negative and uncertain self-view and monitoring their behavior to meet others’ expectations. Presenting new or inauthentic self-versions is challenging as multiple audiences can overlap (Fleming et al., 1990). In online contexts, this problem is amplified as different audiences and social groups (e.g., friends, family, and work colleagues) often occupy the same space, leading to context collapse (van der Nagel & Frith, 2015). Being anonymous may overcome the multiple audience dilemma as a person can strategically adapt their persona to satisfy different audiences without possibly presenting these identities under one self-image. Broadly, this sheds initial light on why some people—but not others—are motivated to seek anonymity and suggests that those with a generally negative self-view may seek anonymity for the gratifications that anonymous environments offer. This may be especially true for those with self-expression motives, as they are additionally self-conscious of how others view them.

#### The Dark Triad

As predicted, Machiavellianism was strongly associated with anonymous self-expression and anonymous toxicity. Machiavellianism is linked to using various self-presentation tactics to maneuver through interpersonal situations and achieve power over others (Bolino & Turnley, 2003; Hart et al., 2019; Rauthmann, 2011), and as such, online anonymity may provide an easier platform to achieve these goals. Against expectations, both anonymous self-expression and toxicity motives were positively correlated with psychopathy and sadism. Although traditionally viewed as having distinctive theoretical roots (Paulhus & Williams, 2002), some have argued that dark personality traits share similarities (Miller et al., 2019). Therefore, these relationships may be due to shared variance between the dark tetrad traits. Indeed, when dark tetrad traits were entered simultaneously in a regression model, the results provided more robust support for our hypothesis, as Machiavellianism is linked with self-expression and toxicity motives. In contrast, psychopathy is only linked to toxic motives.

Finally, narcissism was negatively associated with anonymous self-expression. Although previous research suggests that people high in narcissism favor anonymous environments as they can reach a wider audience (Keipi et al., 2015), we argue that those with narcissistic tendencies may be hesitant to seek anonymity as these environments are unlikely to benefit their reputation, while also inhibiting their ability to receive attention.

#### Personality

We found that people who seek anonymity online—for both toxic and self-expression motives—score lower in honesty-humility, extraversion, agreeableness, and conscientiousness. Elevated dark personality traits are generally predicted by low scores on honesty-humility (Book et al., 2016; Jones & Paulhus, 2017). This aligns with the above-reported findings as dark personality traits were associated with anonymous self-expression and toxic anonymity. Furthermore, as toxic online behavior is socially undesirable, more conscientious and agreeable individuals may be better able to successfully inhibit or avoid these behaviors (McCreery & Krach, 2018). Low extraversion is associated with social anxiety and awkwardness (Stein & Stein, 2008). Consequently, people low in extraversion may be empowered by anonymity due to the ability to reinvent themselves and become socially richer, using the perceived social safety anonymity provides (Amichai-Hamburger & Etgar, 2019).

Toxic anonymity was negatively associated with emotionality and openness to experience. Emotionality is related to fearfulness, anxiety, and dependence on emotional support (Ashton & Lee, 2007). As a result, high emotionality could inhibit motivations to behave toxically in anonymous environments. Likewise, because openness is related to a willingness to accept unconventional people and ideas, people high in openness are unlikely to desire to hurt others for being different (Ashton & Lee, 2007). Interestingly, there was no significant association between anonymous self-expression and emotionality. This prediction was based on previous research that has identified relationships between anxiousness and compensatory internet use (Weidman et al., 2012). However, the lack of an association between emotionality and anonymous self-expression here may indicate that other aspects of emotionality, such as dependency and sentimentality, may not be correlated with motives to self-express while anonymous.^5^

Overall, Study 3b provides a thorough assessment of theoretically relevant individual differences that predicted anonymous online motivations and, in doing so, begins to develop the OAQ’s nomological net. Study 3c aimed to examine how these anonymous motivations predict online behavior.

## Study 3c: Anonymous Motivations and Self-Reported Behavior

In Study 3c, we aimed to assess the predictive validity of the OAQ in relation to self-reported anonymous online behavior. This allows us to potentially use the OAQ to predict people’s behavior in anonymous online environments, depending on their motivations to seek anonymity in the first place. We expected people to engage in behaviors that allow them to achieve their goals. Specifically, we predicted that people who score high on anonymous self-expression motivations would be more likely to engage in benign interpersonal behaviors where they can reveal self-aspects to others and attempt to build or maintain social relationships online (Coyne et al., 2013). Conversely, we expected that people who score high on anonymous toxic motivations be more likely to engage in antisocial interpersonal behaviors, as they can reveal their malevolent self-aspects with little fear of reprisal (Lapidot-Lefler & Barak, 2012; Zimmerman & Ybarra, 2016). We also asked participants questions regarding their online conduct and time spent online.

### Measures

We assessed 11 online behaviors: Lurking, chatting, building relationships, debating, sharing secrets, ghosting (ending a relationship with someone without explanation or communication), being antagonistic, catfishing (luring someone into a relationship with a fictional online persona), trolling, treating people badly, and upsetting other people. Participants were asked how often they engage in each behavior while anonymous online on a four-point scale (1 *= Never*, 4 *= Often*). Participants were also asked if they owned an anonymous social media account and, on average, how many hours per day they spent on the internet for non-work purposes.^6^

### Results

On average, participants spent 5.65 (*SD* = 3.64) hours per day on the internet for non-work purposes. Both anonymous self-expression (*r* = .22, *p* < .001) and toxicity (*r* = .16, *p* = .004) were positively associated with time spent online generally. Anonymous self-expression (*r* = .27, *p* < .001) and toxicity (*r* = .18, *p* = .001) were also positively associated with owning an anonymous social media account.

#### Self-Reported Online Behavior

Because many self-reported online behaviors were assessed in this study, an exploratory factor analysis using Promax rotation and principal axis factoring was conducted to determine whether underlying latent variables may better fit the data. A parallel analysis indicated a two-factor solution should be retained. Subsequently, our two-factor solution explained 40% of the variance with assumptions of sampling adequacy (Kaiser–Myer–Olkin = .86) and sphericity being met χ^2^(55) = 1,164.26, *p* < .001. The first factor—malign behavior—contained five online behaviors considered antisocial and malicious (α = .81). The second factor—benign behavior—included five online behaviors considered positive or not harmful to others (α = .75). See Table C of the supplemental materials for factor loadings. Lurking did not load onto either factor and was subsequently removed from the analysis. We used these two factors to analyze online behavior in this study and Study 4.

Both self-expression and toxicity motives were positively associated with benign and malign behaviors (see Table 7). Next, we ran two multiple regression analyses to assess how the OAQ subscales predict self-reported anonymous online behaviors (Table 9). Anonymous self-expression motives were positively associated with benign behaviors, whereas anonymous toxicity motives were positively associated with malign behaviors. Both predictors showed a medium effect size.

**Table 9:** Multiple Regression Analysis Between the OAQ and Self-Reported Anonymous Online Behavior (Study 3c).

Variable	Benign behavior			Malign behavior		
	*b*	*SE*	*r* [95% CI]	*b*	*SE*	*r* [95% CI]
Intercept	.87	.11		.68	.09	
Anonymous Self-Expression	.26***	.04	0.36 [0.26, 0.45]	.04	.03	0.06 [−0.05, 0.17]
Anonymous Toxicity	.07	.04	0.09 [−0.02, 0.20]	.35***	.03	0.49 [0.37, 0.54]
	*F*(2, 318) = 46.2***			*F*(3, 317) = 82.9***		
	*R*^2^ = .220***			*R*^2^ = .338***		

*Note. N* = 321. Each column represents a separate regression with variables in rows as predictor variables, and variables in the column header as outcome variables. CI = confidence interval.

*** *p* < .001.

### Discussion

In Study 3c, we sought to establish the predictive validity of the OAQ by examining the relationship between motivations to seek anonymity and self-reported anonymous online behavior. We found that a person’s motivation to seek anonymity can predict whether they are likely to engage in social or antisocial behavior while anonymous online. Those with self-expression motives were more likely to report engaging in online behaviors that promote self-presentation and disclosure. In contrast, those motivated by toxicity reported engaging in behaviors that intend to harm others.

More broadly, the results of Study 3b and Study 3c provide insight into the potential goals of people motivated to seek anonymity. People with self-expression motives may use anonymity to achieve self-related goals (e.g., wanting to disclose self-aspects). These goals may stem from a negative and uncertain self-view and an implicit distrust of others. On the contrary, people with toxic motives may use anonymity to achieve other-related goals (e.g., harming others) that, in turn, are self-gratifying as they feed the desires that stem from traits such as sadism and psychopathy. In Study 4, we intend to extend these findings using a daily diary approach.

## Study 4: Diary Study of Self-Reported Online Behavior

Our final study aimed to use a daily diary approach to test whether motivations to seek anonymity were associated with differences in time spent online and self-reported online behavior while both anonymous and identifiable. The benefits of conducting a diary study are that it shortens the interval between the questions and events of interest, thus reducing recall errors, and it uniquely captures human phenomenology (Bolger et al., 2003; Ernala et al., 2020). Our study is the first to evaluate both anonymous and identifiable online behavior through a diary study.

According to UGT, motivations to consume online media influence online behavior (Chan-Olmsted & Wang, 2022). However, motivations are not always identical across multiple forms of media (M.-Y. Chung & Kim, 2015). In our case, anonymous online environments may provide different gratifications to identifiable online environments. Furthermore, people often seek specific environments to pursue their goals. If this pursuit is perceived to be successful, these behaviors may reinforce their motivations for seeking the environment in the first place. Therefore, those who are motivated to seek anonymity should do so to reap the specific gratifications of anonymous online interactions and not online interactions more broadly.

We expected that anonymous motivations would be positively associated with spending time online anonymously. More specifically, we predicted that anonymous self-expression motives would be related to self-reported benign behaviors, and anonymous toxicity motives would be associated with self-reported malign behaviors while anonymous but not while identifiable.

### Method

#### Participants

A power analysis indicated that a sample of 146 participants would provide 80% power with a medium effect size. However, due to the possibility of attrition, we exceeded that number by recruiting 350 university undergraduates who participated for course credit. Twenty-five participants were removed for not completing a minimum of 4 days in the diary study. Therefore, the final sample included 325 participants (261 women, 59 men, 5 non-binary; age range = 16–59, *M* = 20.72, *SD* = 4.72). Of these participants, 51% reported being White, 40% Asian, 1% Hispanic/Latino, and 8% other ethnicities.

#### Measures and Procedures

Please see the supplemental information for a more detailed description of the procedure for Study 4. After providing consent, participants completed an initial baseline survey, including questions about their age, gender, ethnicity, self-esteem, self-concept clarity, Machiavellianism, narcissism, psychopathy, sadism, and the OAQ. Participants then completed a 7-day diary study.

Each day at 9 a.m., participants were sent a short questionnaire asking them to report how much time they spent participating in *active* online behavior while identifiable and anonymous from the previous day (e.g., on Monday, participants reported their behavior for Sunday). We excluded passive online behaviors because they are not visible to other users, meaning that users’ behavior is anonymous to others regardless of whether they intended to be anonymous or identifiable. Therefore, we chose to only focus on active use as the distinction between active use while anonymous and identifiable would be clear to participants. People typically overestimate their time on social media (Verbeij et al., 2021). To minimize this error, we used a seven-point ordinal scale (1 *= 1 to 10 min*, 2 *= 11 to 30 min*, 3 *= 31 to 59 min*, 4 *= 1 to 2 hr*, 5 *= 1 to 3 hr*, 6 *= 3 to 5 hr*, 7 *= more than 5 hr)*. This scale has been shown to reduce error when collecting time spent using social media data via self-report (Ernala et al., 2020). Participants were also asked what behaviors they had engaged in while identifiable and anonymous on the previous day (0 = *no*, 1 = yes). We used the same behaviors from Study 3c, with the addition of posting and the exclusion of lurking, as it is a passive behavior.

### Results

#### Time Spent Online and Individual Differences

Time spent online was assessed using an ordinal scale. We used Spearman’s rank-order correlations to assess time spent online. Both anonymous self-expression (*r_s_* = .25, *p* < .001) and toxicity (*r_s_* = .26, *p* < .001) motives were positively associated with time spent online anonymously and not associated with time spent online while identifiable. We also found that the correlations between the OAQ and the reported individual differences were mostly consistent with the results of Study 3b (see Tables 6 and 7). However, in Study 4, toxic anonymity was positively associated with narcissism and not with self-esteem.

#### Self-Reported Online Behavior

Initially, we created measures of benign and malign behavior by summing the behaviors across the seven days. However, due to low event rates for some behaviors, we made a binomial measure of benign and malign self-reported online behaviors engaged in when identifiable and anonymous. Specifically, we scored these variables as 0 for *no relevant behaviors* and 1 for *one or more relevant behaviors*. Anonymous self-expression was positively associated with anonymous benign behaviors. Anonymous toxicity was positively related to anonymous and identifiable malign behaviors, anonymous benign behaviors, and negatively associated with identifiable benign behaviors (see Table 7).

A series of logistic regressions were conducted to assess how—when entered simultaneously—the OAQ subscales predict whether participants reported engaging in benign or malign online behaviors (see Table 10). Anonymous self-expression was positively associated with anonymous benign behavior. More specifically, we found that with every unit increase of anonymous self-expression motives, participants were 87% more likely to engage in anonymous benign behaviors. Anonymous toxicity was positively associated with identifiable and anonymous malign behaviors and negatively related to identifiable benign behaviors. This means that for every unit increase of anonymous toxicity motives, participants were 315% more likely to engage in malign behaviors when anonymous, 140% more likely to engage in malign behaviors when identifiable, and 56% less likely to engage in benign behaviors when identifiable.

**Table 10:** Logistic Regression of Anonymous Motivations Predicting Self-Reported Anonymous/Identifiable Benign/Malign Behavior Across a Seven-Day Diary Study

Variable	Anonymous						Identifiable					
	Benign			Malign			Benign			Malign		
	β	Wald χ^2^	OR [95%CI]	β	Wald χ^2^	OR [95%C]I	β	Wald χ^2^	OR [95%CI]	β	Wald χ^2^	OR [95%CI]
(Intercept)	−2.23***	17.9	0.11 [0.04, 0.29]	−7.47***	15.4	0.01 [0.01, 0.02]	5.69***	16.6	296.32 [24.30, 6032.91]	−4.27***	19.8	0.01 [0.01, 0.06]
Anonymous Self-Expression	0.63***	12.2	1.87 [1.33, 2.69]	0.11	0.03	1.12 [0.33, 4.02]	−0.21	0.20	0.91 [0.34, 2.02]	−0.04	0.01	1.04 [.51, 2.16]
Anonymous Toxicity	0.08	0.20	1.09 [0.76, 1.57]	1.42**	7.20	4.15 [1.53, 12.67]	−0.81*	4.0	0.44 [0.19, 0.98]	0.88**	6.80	2.41 [1.26, 4.76]

*Note. N* = 325. Values in brackets next to OR values indicate a 95% confidence interval. Each column represents a separate logistic regression with variables in rows as predictor variables, and variables in the column header as outcome variables. OR = Odds Ratio; CI = confidence interval.

* *p* < .05. **p < .01. ***p < .001.

### Discussion

We again found that motivations to seek anonymity online were associated with differences in self-reported anonymous online behavior. People who were motivated to seek anonymity spent more time online anonymously than identifiably. Furthermore, the behaviors engaged were specific to the motivations. Those higher in anonymous self-expression motives were more likely to engage in benign behaviors only in anonymous environments. This supports a UGT approach, as the gratifications sought by people with specific anonymous motivations were associated with behaviors that need to be enacted to obtain these gratifications. Presumably, this is because anonymous online environments allow them to attain their sought gratifications.

Those higher in toxic motives were more likely to engage in malign behaviors across both anonymous and, to a lesser extent, identifiable online spaces. This indicates that although anonymity may reduce the ramifications of engaging in toxic behavior, their desire to behave nefariously also spreads to identifiable environments. Unexpectedly, people with toxic motives were less likely to engage in benign behaviors while identifiable. Malign behaviors likely come with a reputational cost. Therefore, engaging in these behaviors while identifiable may mean that others do not want to engage in benign behaviors, such as chatting or building relationships.

Finally, Study 4 replicated the previously observed relationships between the OAQ subscales and dark tetrad traits, increasing confidence in the robustness and reliability of these relationships. Similarly, the relationships between self-esteem, self-concept clarity, and anonymous self-expression motives were consistent with Study 3b. However, unlike in Study 3b, self-esteem was not associated with anonymous toxicity motives.

## General Discussion

Online environments have come a long way from “computer conferencing and electronic mail systems” (Lea et al., 1992, p. 90), and there are now more opportunities to seek anonymity online. Based on the SIDE model, previous research has found that anonymous situations often result in deindividuation, increasing feelings of group salience, and polarization of group norms (Lea & Spears, 1991; Postmes & Spears, 2002). However, fewer studies have aimed to understand *why* some people are motivated to interact in anonymous online environments. It is important to consider that individuals seek environments that allow them to meet their goals. Thus, it is not simply the power of the situation that explains behavior but the predisposing traits and motivations that draw individuals to those situations. Being anonymous has advantages, and although some people are indifferent to its potential gratifications, others actively seek it out. Across four studies, we argued and demonstrated differences in people’s motivations to seek anonymity, with different dispositional factors presumably driving these effects. We also showed how these anonymous motivations predict differences in how people behave online, both anonymously and identifiably. We discuss these results and their implications in detail below.

### Motivations to Seek Anonymity

We aimed to synthesize past research and understand the motivations for seeking anonymity. To do so, we developed and validated a measure of motivations to seek online anonymity in Studies 1, 2, and 3a. First, we found that some people are motivated to seek anonymity as a means of self-expression. People who scored high on this subscale indicated that they felt more comfortable interacting with others and experimenting with various self-aspects while anonymous. The anonymous self-expression subscale combined items relating to both self-disclosure and self-presentation strategies. Although distinct constructs, the two are not mutually exclusive, as self-presentation can include disclosing true information (Schlenker & Wowra, 2003).

The desire to self-express and have interpersonal connections are fundamental human motivations, and as such, a perceived benefit of being anonymous online is it can help people present and express themselves more easily and in separate ways from how they would self-express while identifiable (McKenna et al., 2002; Sprecher et al., 2013). Therefore, some people seek anonymity to gratify their need to self-express.

Second, we found that some people use anonymity as a tool to engage in toxic or antisocial behavior. Whereas anonymity can be self-beneficial, it can amplify antisocial behaviors more problematically as people often remain unaccountable for their actions. However, this lack of accountability benefits their goal of behaving nefariously toward others, and therefore they seek anonymity to gratify their need to act toxically. Therefore, our first contribution to the literature when assessing anonymous motivations is validating a reliable measure of different motivations for why people seek anonymity online.

### Anonymous Motivations and Individual Differences

The second phase of our research aimed to identify theoretically relevant individual differences that predict people’s motivations to seek anonymity online. Notably, seeking anonymity was associated with an uncertain self-view. Self-concept clarity is positively associated with well-being (Usborne & Taylor, 2010), and as such, people strive to attain a clear sense of who they are (Aron et al., 2000). However, those who lack a clear sense of who they are often avoid adjusting their self-concept, due to the risk of self-confusion, or judgment from others (Emery et al., 2015). Irrespective of one’s goals, anonymous environments provide a space where people can experiment with who they are without fear of repercussion. This may be especially important for people who lack a strong sense of self or are socially inhibited (Forest & Wood, 2012; Lee, 2009; McKenna et al., 2002). Motives to seek anonymity were also typified by Machiavellian tendencies. Machiavellians are strategic and will only exploit others when there is little chance of reprisal. As anonymity often mitigates the possibility of social consequences, those high in Machiavellianism seek it out as it is often the most strategic option for achieving their goals.

Individual differences predict overall motives to seek anonymity, but our research also uncovered differences in how dispositional factors predict each motivation. Anonymous self-expression was typified by low self-esteem and self-consciousness, whereas toxic motives were consistently associated with dark personality traits such as psychopathy and sadism. Therefore, the results indicate that, although similar, self-expression and toxicity motives are conceptually distinct in some respects.

### Anonymous Motivations and Online Behavior

Beyond showing how individual differences predict motivations to seek anonymity, the third phase of our research provided evidence for how these motivations predict self-reported online behavior. Both toxic and self-expression motivations predicted spending more time online anonymously but not more time while identifiable. Furthermore, we found that anonymous self-expression motives were associated with benign behaviors that promote self-expression, whereas toxic motivations were related to engaging in malevolent online behaviors.

These results have important implications for research into anonymous online behavior. People seek anonymity to obtain the perceived gratifications on offer, including, but not limited to, remaining unaccountable for one’s actions and potentially accentuating the salience of group norms for specific communities (Chan et al., 2022; Clark-Gordon et al., 2019). People enact specific behaviors—either benign or malign—that allow them to reap the benefits of the gratifications they seek. For example, it is not solely that people who find themselves in anonymous situations end up trolling because of the effects of deindividuation and social norms. Rather, people who enjoy antagonizing others know that this is easier to achieve in anonymous environments, so they actively choose those places. Importantly, this research shows that the distinction between anonymous and identifiable online environments is novel and indicates that our research is not merely a recontextualization of online behavior research more broadly.

### Implications, Limitations, and Future Directions

From the early 1980s, ten years before the World Wide Web was accessible, people have argued that online anonymity leads to the proliferation of uncivil and manipulative behaviors and should be controlled and restricted (Siegel et al., 1986). Even today, tech giants and world leaders continue to call for using “real names” on the internet (van der Nagel & Frith, 2015). Others, however, argue that anonymity is essential to privacy and free speech in the online world (Hogan, 2013). One implication of our research is that the benefits of anonymity stretch beyond privacy and free speech. These environments may allow people to express certain self-aspects better while giving people a platform to behave antisocially with less concern about potential negative repercussions. Although anonymity abets antisocial behavior, understanding the dispositional and motivational factors that draw people to anonymous environments illustrates the significant role of predisposing factors. As such, we suggest future research should investigate how users can best reap the potential benefits that anonymous spaces can offer while also being aware of the increased risk of antisocial behavior by others.

Our research also holds implications for broader theories of anonymity. Decades of psychological research have focused on the situational and contextual factors influencing behavior in anonymous environments. Before the internet was widely available, situations allowing for anonymous behavior were not necessarily common or sought out. However, technology has progressed, and the possibility of being anonymous online is now available to most people. We have demonstrated the importance of exploring the perceived benefits of anonymity through a uses and gratifications approach. In doing so, we investigated why people are motivated to seek anonymity to gratify their specific needs and use anonymous environments to pursue self- or other-related goals. The nature of the internet and how people interact in these online environments have shifted. Our understanding of anonymous environments—and online environments more broadly—is essential to continually develop and grow to account for the added complexity this brings. Therefore, we argue that future research should aim to combine these two approaches and use an integrative, interactionist perspective to investigate anonymous online behavior further.

Of course, these results are not without limitations. Our samples predominantly represented Western populations, and we expect our results to be broadly generalizable across WEIRD cultures (Simons et al., 2017). However, we do not have evidence that these findings will occur cross-culturally. The online world is global and far-reaching, and previous work has found cultural differences in internet behavior (Kim et al., 2011). It is therefore conceivable that anonymous motivations also differ cross-culturally, meaning this would be an exciting avenue for future research to investigate. A second limitation of our study is that not all items in the original scale explicitly mentioned online behavior. Although we conducted an additional study indicating that not explicitly mentioning the online nature of the scale did not influence the results, we recommend that researchers who plan to use the OAQ in future studies should use the revised items in Appendix to reduce potential ambiguity and allow the items to be used in a multiscale survey.

A final limitation of our research is the use of correlational and cross-sectional methods. Cross-sectional data allow us to assess the complexity of anonymous motivations by addressing multiple variables. However, with cross-sectional research, the possible conclusions we can draw depend on the predictors we have included. The causal role of intervening variables cannot be determined, as alternative theoretical models cannot be ruled out. Therefore, future research should implement design features such as experimental manipulation with random assignment or longitudinal research to better tease apart potential causal pathways. Furthermore, future investigations should continue to expand our understanding of why people seek anonymity by assessing prosocial or altruistic behaviors (e.g., donating anonymously to an online charity; Raihani, 2014).

## Conclusion

It appears that Oscar Wilde was correct in his assertions regarding why people often seek anonymity. Our research indicates that motivations to seek anonymity vary and relate to differences in views of the self, personality, and online behavior. Specifically, people seek anonymity to pursue self- or other-related goals, with motivations differing depending on the goals they wish to achieve. For decades, research into anonymity has been dominated by situational and context-based approaches, and we believe a more robust interactionist perspective is necessary when investigating anonymous online spaces. In this paper, we have validated a reliable new measure to assess motivations to be anonymous and identified associated individual differences and behaviors. We hope our research sparks renewed interest in anonymity and facilitates further advances in understanding the gratifications of online environments.

## Supplemental Material

sj-docx-1-psp-10.1177_01461672231210465 – Supplemental material for Why Do People Sometimes Wear an Anonymous Mask? Motivations for Seeking Anonymity Online Supplemental material, sj-docx-1-psp-10.1177_01461672231210465 for Why Do People Sometimes Wear an Anonymous Mask? Motivations for Seeking Anonymity Online by Lewis Nitschinsk, Stephanie J. Tobin, Deanna Varley and Eric J. Vanman in Personality and Social Psychology Bulletin

sj-docx-2-psp-10.1177_01461672231210465 – Supplemental material for Why Do People Sometimes Wear an Anonymous Mask? Motivations for Seeking Anonymity Online Supplemental material, sj-docx-2-psp-10.1177_01461672231210465 for Why Do People Sometimes Wear an Anonymous Mask? Motivations for Seeking Anonymity Online by Lewis Nitschinsk, Stephanie J. Tobin, Deanna Varley and Eric J. Vanman in Personality and Social Psychology Bulletin

## Appendix

### The Online Anonymity Questionnaire

This questionnaire is focused on why people are sometimes anonymous on the internet. Please respond to the statements below by indicating how much you agree/disagree with each.

1. I feel more comfortable disclosing information about my ideas, thoughts, and feelings when I am anonymous *online*.
2. Being anonymous *online* allows me to share thoughts and feelings I otherwise would not share with people who know me.
3. Being anonymous *online* allows me to experiment with new ideas.
4. I feel like I can be somebody else when I am anonymous *online*.
5. I can present myself in a different way when I am anonymous *online*.
6. Being anonymous online allows me to escape or distract myself from reality.
7. When I am anonymous online, I can talk to people who would not normally talk to me in the offline world.
8. Being anonymous *onlin*e allows me to join groups I would not normally join in the real world.
9. I can connect with people I normally would not when I’m anonymous *online*.
10. I feel like I can express my true self when I am anonymous *online.*
11. I am more likely to do things that are unlawful or illegal when I am anonymous *online*.
12. When I am anonymous *online*, I do things that are normally unacceptable in society.
13. I get satisfaction from aggravating people anonymous online.
14. Being anonymous online is fun because I don’t get in trouble for what I say.
15. When I am anonymous *online*, I find it easier to trick and manipulate people.
16. I like being anonymous *online* because I can say whatever I want without consequences.

### Scoring Procedure

Anonymous Self-Expression: 10 Items

1, 2, 3, 4, 5, 6, 7, 8, 9, 10

Anonymous Toxicity: 6 Items

11, 12, 13, 14, 15, 16

Scale Ranges from 1 (strongly disagree) to 5 (strongly agree).

### Note

Italicized words were not included in the version of OAQ used within this manuscript. We recommend researchers who plan to use the OAQ in future studies include the italicized words to reduce potential ambiguity and allow the items to be used in a multiscale survey.

## Notes

1. Additional data from this sample are presented in a separate empirical report (Nitschinsk et al., 2022b). This report is based on the same dataset but investigates different research questions.

2. In Study 2, ten additional items were originally included in the OAQ to assess a third factor. This factor was ultimately removed as it was conceptually similar to the other factors in the scale. These 10 items are not reported in the manuscript but available in the supplementary materials.

3. Although the OAQ is preceded with information that the scale is in relation to online anonymity, not all items explicitly mention online behavior. This is a potential limitation as some readers might read into the different item formulations. We ran an additional study (*N* = 319) where all items were rewritten to explicitly mention that the item is pertaining to online behavior. Both the anonymous self-expression and anonymous toxicity subscales remained internally consistent, and the overall model fit was acceptable. This indicates that not explicitly mentioning the online nature of the scale within each item has not influenced the results of the manuscript. Nonetheless, we advise researchers who plan to use the OAQ in future studies should use the updated version of the scale. See supplemental materials for more information.

4. Descriptive statistics and scale reliabilities for the POSSA and the Dark Triad are presented in a separate brief report (Nitschinsk et al., 2022a). This brief report is based on the same dataset but investigates different research questions.

5. Correlation analysis between anonymous self-expression and facet-level traits of emotionality shows that anonymous self-expression is positively associated with anxiety (*r* = .16, *p* = .005) and fearfulness (*r* = .16, *p* = .003), negatively associated with sentimentality (*r* = -.19, *p* = .001) and not associated with dependence (*r* = .00, *p* = .998).

6. In Study 3 and 4 we additionally collected data relating to the social media websites participants use while anonymous. Due to the manuscript’s length, we deviated from our original preregistration and did not include this data in the final manuscript.
