# Supplementary material for: Why Do People Sometimes Wear an Anonymous Mask? Motivations for Seeking Anonymity Online
Source: Pers Soc Psychol Bull. 2023 Nov 24;51(7):1099–120. doi: 10.1177/01461672231210465 (PMC12130607; doi:10.1177/01461672231210465)
Supplement: sj-docx-1-psp-10.1177_01461672231210465 – Supplemental material for Why Do People Sometimes Wear an Anonymous Mask? Motivations for Seeking Anonymity Online [file sj-docx-1-psp-10.1177_01461672231210465.docx]

Why Do People Sometimes Wear an Anonymous Mask?

Motivations for Seeking Anonymity Online

**Codebook**

This codebook explains the column names for all datasets included in the manuscript. Markdowns for data cleaning and analysis are found on the OSF.

**Study 1: Development of the Online Anonymity Questionnaire**

| Column Name | Column Meaning |
| --- | --- |
| subjectid | Participant ID |
| Gender | Male = 1  Female = 2  Other = 6 |
| Birth | 1 = 2001, 2 = 2000… 95 = 1907 etc. |
| Language | 1 = English  2 = Spanish  3 = Chinese  4 = French  5 = German  6 = Dutch  7 = Japanese  8 = Hebrew  9 = Swedish  10 = Other (specify) |
| Education | 1 = Less than High School  2 = High School or Equivalent  3 = Some University/College  4 = University/College Degree  5 = Masters Degree  6 = Doctoral Degree (PhD)  7 = Professional Degree (JD, MD) |
| Q47 – Relationship Status | 1 = Single  2 = Dating  3 = In a committed relationship  4 = Married/de facto |
| Q25_1: Q25_8, Q25_11, Q25_15, Q25_16, Q25_21, Q25_23, Q25_25 | Online Anonymity Questionnaire – Self-Expression Items |
| Q25_12: Q25_14, Q25_26:Q25_30 | Online Anonymity Questionnaire – Toxicity Items |
| Q25_17, Q25_19, Q25_24 | Online Anonymity Questionnaire – Indifference Items |

**Study 2: Refinement of the Online Anonymity Questionnaire**

| Column Name | Column Meaning |
| --- | --- |
| Prolific ID | Participant ID |
| Gender | Male = 51  Female = 52  Other = 53 |
| Nationality | 192 countries.  404 = Afghanistan  Zimbabwe = 596.  Notably:  United States of America = 588  United Kingdom = 586  Canada = 434  Australia = 412 |
| Age | Text entry. |
| noaq_1:noaq_6, noaq_9:noaq_12 | Online Anonymity Questionnaire – Self-Expression Items |
| noaq_13, noaq_14, noaq_16, noaq_18, noaq_20, noaq_21 | Online Anonymity Questionnaire – Toxicity Items |
| noaq_22, noaq_24:noaq_30 | Online Anonymity Questionnaire – Indifference Items |

**Study 3: Scale Validation OAQ**

| **Column Name** | **Column Meaning** |
| --- | --- |
| prolificid | Participant ID |
| gender | Gender – Male = 1  Female = 2  Non-Binary = 3  Prefer not to say = 4  Prefer to self-describe = 5 |
| ethnicity | 1 = White  2 = Asian  3 = African/African American  4 = Hispanic/Latino  5 = Other (specify) |
| age | Text entry. |
| language | 1 = English  2 = Mandarin  3 = Arabic  4 = Cantonese  5 = Vietnamese  6 = Spanish  7 = Japanese  8 = Hindi  9 = Punjabi  10 = Other (specify) |
| Education | 1 = Less than High School  2 = High School or Equivalent  3 = Some University/College  4 = University/College Degree  5 = Masters Degree  6 = Doctoral Degree (PhD)  7 = Professional Degree (JD, MD) |
| relationship | 1 = Single  2 = Dating  3 = In a committed relationship  4 = Married/de facto |
| noaq_1:noaq_6, noaq_9:noaq_12 | Online Anonymity Questionnaire – Self-Expression Items |
| noaq_13, noaq_14, noaq_16, noaq_18, noaq_20, noaq_21 | Online Anonymity Questionnaire – Toxicity Items |
| noaq_22, noaq_24:noaq_30 | Online Anonymity Questionnaire – Indifference Items |
| poss_1:poss_21 | Presentation of Online Self Scale |
| sdt_1:sdt_27 | Short Dark Triad Scale |
| ssis_1:ssis_10 | Short Sadistic Impulse Scale |
| scsr_1:scsr_22 | Self-Consciousness Scale Revised |
| sms_1:sms_13 | Self-Monitoring Scale |
| scc_1:scc_12 | Self-Concept Clarity |
| sesteem_1:sesteem_10 | Self-Esteem |
| hexaco_1:hexaco_60 | HEXACO |
| chat1, antagonistic1, debate1, lurk1, relation1, mean1, catfish1, ghost1, troll1, secret1, treatbad1, upset1 | Online Behaviors – How much time do you spend engaging in these online behaviors?  1 = Never  2 = Rarely  3 = Sometimes  4 = Often |
| chat2, antagonistic2, debate2, lurk2, relation2, mean2, catfish2, ghost2, troll2, secret2, treatbad2, upset2 | What social media sites do you use to engage with X behavior?  1 = Facebook  2 = Instagram  3 = Twitter  4 = YouTube  5 = Reddit  6 = TikTok  7 = online dating apps  8 = online forums  9 = multiplayer online gaming  10 = twitch  11 = 4chan  12 = other. |
| screen 1 – do you have a fake social media for professional reasons?  screen 2 – do you have a fake social media account?  screen 3 – do people who follow you know who you are?  screen 4 – how many hours a day do you use the internet for non-work purposes?  screen 5 – what do you consider anonymity | Screen 1, 2, and 3: 1 = yes, 2 = no  Screen 4 = 1 – 19 Sliding Scale (hours)  Screen 5 = Textbox |

**Study 4: Diary Study**

Note: This codebook is for the clean data set for Study 4. To view how this dataset was cleaned, see the r-markdown titled “OAQ_Study4_Markdown_Prepping”.

| **Column Name** | **Column Meaning** |
| --- | --- |
| id | Participant ID |
| df1$gender | Gender – Male = 1  Female = 2  Non-Binary = 3  Prefer not to say = 4  Prefer to self-describe = 5 |
| df1$age | Text Entry |
| df1$ethnicity | 1 = White  2 = Asian  3 = African/African American  4 = Hispanic/Latino  5 = Other (specify) |
| oaqself | Anonymous Self-Expression – Beginning of week |
| oaqtoxic | Anonymous Toxicity – Beginning of week |
| oaqindifferent | Anonymity Indifferent – Beginning of week |
| oaqelf2 | Anonymous Self-Expression - End of week |
| oaqtoxic2 | Anonymous Toxicity - End of week |
| oaqindifferent2 | Anonymity Indifference - End of week |
| scc | Self-concept clarity |
| sesteem | Self-esteem |
| sdtn | Narcissism |
| sdtm | Machiavellianism |
| sdtp | Psychopathy |
| ssismeans | Sadism |
| smuglobal | Time spent online identifiably on average over the week. |
| smuaglobal | Time spent online anonymously on average over the week. |
| smuglobalmed | Median time spent online identifiably over the week. |
| smuaglobalmed | Median time spent online anonymously over the week. |
| smuchoice | How many days in the week did you choose to use social media identifiably |
| smuachoice | How many days in the week did you choose to use social media anonymously |
| smuCHAT, smuSECRET, smuDEBATE,smuRELATIONSHIP, smuGHOST, smuPOST, smuANTAG, smuCAT, smuTROLL, smuTBAD, smuUPSET | How many days in the week did you do X behaviour on social media while identifiable |
| smuaCHAT, smuaSECRET, smuaDEBATE, smuaRELATIONSHIP, smuaGHOST, smuaPOST, smuaANTAG, smuaCAT, smuaTROLL, smuaTBAD, smuaUPSET | How many days in the week did you do X behaviour on social media while anonymous |
| smubenign2, smumalign2, smuabenign2, smuamalign2 | Number of benign/malign behaviors engaged in while anonymous/identifiable over a week.   - This was later converted in a binomial measure, see R-Markdown. |

**Study 5: Additional Study Test-Retest and Expanding the Nomological Net**

| **Column Name** | **Column Meaning** |
| --- | --- |
| PROLIFIC_PID | Participant ID |
| gender | Gender – Male = 1  Female = 2  Non-Binary = 3  Prefer not to say = 4  Prefer to self-describe = 5 |
| ethnicity | 1 = White  2 = Asian  3 = African/African American  4 = Hispanic/Latino  5 = Other (specify) |
| age | Text entry. |
| language | 1 = English  2 = Mandarin  3 = Arabic  4 = Cantonese  5 = Vietnamese  6 = Spanish  7 = Japanese  8 = Hindi  9 = Punjabi  10 = Other (specify) |
| Education | 1 = Less than High School  2 = High School or Equivalent  3 = Some University/College  4 = University/College Degree  5 = Masters Degree  6 = Doctoral Degree (PhD)  7 = Professional Degree (JD, MD) |
| relationship | 1 = Single  2 = Dating  3 = In a committed relationship  4 = Married/de facto |
| oaq_1 : oaq_10 | Online Anonymity Questionnaire – Self-Expression Items |
| oaq_11 : oaq_16 | Online Anonymity Questionnaire – Toxicity Items |
| oaq_17 : oaq_24 | Online Anonymity Questionnaire – Indifference Items |
| fnes_1 : fnes_13 | Fear of Negative Evaluation Scale |
| loneliness_1 : loneliness_20 | Loneliness Scale |
| twomach_1 : twomach_12 | Machiavellianism two-dimensional scale |
| sdt_1 : sdt_27 | Short Dark Triad Scale |
| ssismeans_1 : ssismeans_10 | Sadism |
| scc_1:scc_12 | Self-concept clarity |
| sesteem_1 : sesteem_10 | Self-esteem |
| scenario1_1 | Scenario asking if participants would choose to make an anonymous or identifiable social media profile.  -5 = Completely Anonymous, 5 = Completely Identifiable |
| scenario2_1 | Scenario asking if participants would choose to participate in a moderated or unmoderated forum.  -5 = No Moderation, 5 = Heavy Moderation |
| Test-retest | The .csv file title “OAQ_Study5_TestRetestMatched” includes the mean scores for all personality characteristics, along with the OAQ administered at time 1, and administered at time 2.  oaq_1 : oaq_24 = time 1  oaq2_1 : oaq2_24 = time 2 |
