# Supplementary material for: Why Do People Sometimes Wear an Anonymous Mask? Motivations for Seeking Anonymity Online
Source: Pers Soc Psychol Bull. 2023 Nov 24;51(7):1099–120. doi: 10.1177/01461672231210465 (PMC12130607; doi:10.1177/01461672231210465)
Supplement: sj-docx-2-psp-10.1177_01461672231210465 – Supplemental material for Why Do People Sometimes Wear an Anonymous Mask? Motivations for Seeking Anonymity Online [file sj-docx-2-psp-10.1177_01461672231210465.docx]

**Supplemental Materials**

**Study 1**

***The Online Anonymity Questionnaire Study 1: Initial Item Generation***

This questionnaire is focused on *why* people are sometimes **anonymous on the Internet**. Please respond to the statements on anonymity below by indicating how much you agree/disagree with each.

1. I feel more comfortable disclosing information about my ideas, thoughts, and feelings when I am anonymous.
2. I feel a sense of power when I am anonymous.
3. Being anonymous allows me to share thoughts and feelings I otherwise wouldn’t share with people who know me.
4. Being anonymous allows me to experiment with new ideas.
5. I feel like I can be someone else when I’m anonymous.
6. I can present myself in a different way when I’m anonymous.
7. Being anonymous while online is fun.
8. Using anonymous online identities allows me to escape or distract myself from reality.
9. I enjoy sharing opposing opinions anonymously, even for topics I am sometimes not passionate about.
10. I feel less responsible for what I do and say online when I am anonymous.
11. I feel like I can say things I normally wouldn’t when I am anonymous.
12. I am more likely to do things that are unlawful or illegal when I am anonymous.
13. When I’m anonymous I do things that are normally unacceptable in society.
14. I feel more afraid of sharing creative ideas when I’m anonymous.
15. Being anonymous allows me to join groups I wouldn’t normally belong to in the real world.
16. I can connect with people I normally wouldn’t when I’m anonymous.
17. Being anonymous is no more fun than in real life.
18. I seek close connections with other people when I’m anonymous.
19. I don’t express myself any better when I’m anonymous.
20. I’m always my true self when I’m anonymous.
21. I feel safer online when I’m anonymous.
22. I enjoy acting out different identities when I’m online.
23. When I am anonymous online, I can talk to people who wouldn’t normally talk to me in the offline world.
24. When I’m anonymous online, I am no more honest than I am in the real world.
25. I feel like my privacy is protected when I’m anonymous.
26. I get satisfaction from aggravating people anonymously online.
27. I am more likely to be anonymous online when I am bored.
28. I can use anonymous platforms to find people who will agree with my ideas.
29. I have sometimes been anonymous online to express my anger.
30. I think being anonymous online could allow me to be famous.

**Table A**
*Exploratory Factor Analysis for Study 1 using Promax Rotation and Principal Axis Factoring.*

| Name | Factor 1 | Factor 2 | Factor 3 |
| --- | --- | --- | --- |
| 1. I feel more comfortable disclosing information about my ideas, thoughts and feelings when I am anonymous. | .798 | -.206 |  |
| 1. I feel a sense of power when I am anonymous. | .679 |  |  |
| 1. Being anonymous allows me to share thoughts and feelings I otherwise wouldn’t share with people who know me. | .812 |  |  |
| 1. Being anonymous allows me to experiment with new ideas. | .794 |  |  |
| 1. I feel like I can be someone else when I’m anonymous. | .710 |  |  |
| 1. I can present myself in a different way when I’m anonymous. | .826 |  |  |
| 1. Being anonymous while online is fun. | .788 |  |  |
| 1. Using anonymous online identities allows me to escape or distract myself from reality. | .551 | .234 |  |
| 1. I enjoy sharing opposing opinions anonymously, even for topics I am sometimes not passionate about. | .280 | .324 |  |
| 1. I feel less responsible for what I do and say online when I am anonymous. | .313 | .265 |  |
| 1. I feel like I can say things I normally wouldn’t when I am anonymous. | .589 | .162 | -.146 |
| 1. I am more likely to do things that are unlawful or illegal when I am anonymous. |  | .595 |  |
| 1. When I’m anonymous I do things that are normally unacceptable in society. |  | .696 |  |
| 1. I feel more afraid of sharing creative ideas when I’m anonymous. | -.295 | .540 | .241 |
| 1. Being anonymous allows me to join groups I wouldn’t normally belong to in the real world. | .558 | .230 |  |
| 1. I can connect with people I normally wouldn’t when I’m anonymous. | .629 | .115 |  |
| 1. Being anonymous is no more fun than in real life. |  | .133 | .504 |
| 1. I seek close connections with other people when I’m anonymous. | .128 | .325 | .230 |
| 1. I don’t express myself any better when I’m anonymous. | -.149 |  | .498 |
| 1. I’m always my true self when I’m anonymous. | .119 | -.299 | .405 |
| 1. I feel safer online when I’m anonymous. | .734 | -.115 | .228 |
| 1. I enjoy acting out different identities when I’m online. | .240 | .385 | -.193 |
| 1. When I am anonymous online, I can talk to people who wouldn’t normally talk to me in the offline world. | .536 | .185 |  |
| 1. When I’m anonymous online, I am no more honest than I am in the real world. | .152 |  | .394 |
| 1. I feel like my privacy is protected when I’m anonymous. | .631 | -.114 |  |
| 1. I get satisfaction from aggravating people anonymously online. |  | .697 | -.115 |
| 1. I am more likely to be anonymous online when I am bored. | .114 | .559 | -.137 |
| 1. I can use anonymous platforms to find people who will agree with my ideas. | .225 | .429 |  |
| 1. I have sometimes been anonymous online to express my anger. |  | .639 |  |
| 1. I think being anonymous online could allow me to be famous. |  | .693 | .112 |

*Note.* Factor loadings reflect values from the pattern matrix. Bolded coefficients denote primary factor loadings. Loadings < .10 were suppressed from the table. *N* = 243

**Item Changes from Study 1 to Study 2**

**Factor 1: Anonymous Self-Expression**

From Study 1, the first factor *Anonymous Self-Expression* included 14 items that were generally associated with motivations to self-expression, disclose, or present different self-aspects while anonymous online. From the first study, anonymous self-expression is potentially the most well-defined factor, however, we believe improvements and refinements were necessary. First, the items *I feel a sense of power when I’m anonymous* and *being anonymous while online is fun* are both vague in the motivation they are seeking to address. While both items loaded strongly onto the factor it was decided to remove these items as they did not address the same construct as other items in the factor and were therefore removed. Second, two items in the factor assess online privacy concerns, which we decided to remove from the scale. The reasoning behind the first item *I feel safer online when I’m anonymous* was we felt it addressed two separate motivations. The first was a motivation to seek anonymity as you feel emotionally safe, which does address the self-expression construct. The item however also addresses general safety, or privacy concerns and therefore these two possible explanations make the item vague in what it’s attempting to represent. The second item, *I feel like my privacy is protected when I’m anonymous* more directly assesses online privacy concerns, but as this item does not reflect the factors construct it was removed. Two items were also rewritten for clarity.

***Initial Items from the First Exploratory Factor Analysis***

1. I feel more comfortable disclosing information about my ideas, thoughts, and feelings when I am anonymous.
2. I feel a sense of power when I am anonymous. (Removed)
3. Being anonymous allows me to share thoughts and feelings I otherwise wouldn’t share with people who know me.
4. Being anonymous allows me to experiment with new ideas.
5. I feel like I can be someone else when I’m anonymous.
6. I can present myself in a different way when I’m anonymous.
7. Being anonymous while online is fun. (Removed)
8. Using anonymous online identities allows me to escape or distract myself from reality. (Rewritten)
9. I feel like I can say things I normally wouldn’t when I am anonymous. (Rewritten)
10. Being anonymous allows me to join groups I wouldn’t normally belong to in the real world.
11. I can connect with people I normally wouldn’t when I’m anonymous.
12. I feel safer online when I’m anonymous. (Removed)
13. When I am anonymous online, I can talk to people who wouldn’t normally talk to me in the offline world.
14. I feel like my privacy is protected when I’m anonymous. (Removed)

***Item Generation for the Second Exploratory Factor Analysis***

1. I feel more comfortable disclosing information about my ideas, thoughts, and feelings when I am anonymous.
2. Being anonymous allows me to share thoughts and feelings I otherwise wouldn’t share with people who know me.
3. Being anonymous allows me to experiment with new ideas.
4. I feel like I can be somebody else when I am anonymous.
5. I can present myself in a different way when I’m anonymous.
6. Being anonymous online allows me to escape or distract myself from reality. (Rewritten)
7. When I am anonymous online, I can talk to people who wouldn’t normally talk to me in the offline world.
8. Being anonymous allows me to join groups I wouldn’t normally join in the real world.
9. I can connect with people I normally wouldn’t when I’m anonymous.
10. I feel like I can express my true self when I am anonymous. (Rewritten)

**Factor 2: Toxic Anonymity**

From Study 1, the second factor *Toxic Anonymity* included eight items generally associated with motivations to behave toxically while online. While five of the items in the factor directly reflected toxic or antisocial behaviour, two items (5 and 6) were indirect reflections of online toxicity, while finally item three did not reflect the construct. Furthermore, these three items showed the lowest factor loadings, and highest cross loadings. Therefore, it was decided for these three items to be removed, and four new items were included to better represent motivations to behave toxically while anonymous. Finally, one item was rewritten to improve clarity.

***Initial Items from the First Exploratory Factor Analysis***

1. I am more likely to do things that are unlawful or illegal when I am anonymous.
2. When I’m anonymous I do things that are normally unacceptable in society.
3. I feel more afraid of sharing creative ideas when I’m anonymous. (Removed)
4. I get satisfaction from aggravating people anonymously online.
5. I am more likely to be anonymous online when I am bored. (Removed)
6. I can use anonymous platforms to find people who will agree with my ideas. (Removed)
7. I have sometimes been anonymous online to express my anger. (Rewritten)
8. I think being anonymous online could allow me to be famous.

***Item Generation for the Second Exploratory Factor Analysis***

1. I am more likely to do things that are unlawful or illegal when I am anonymous.
2. When I’m anonymous I do things that are normally unacceptable in society.
3. Being anonymous online makes me feel lonelier. (New)
4. I get satisfaction from aggravating people anonymously online.
5. I think being anonymous could allow me to be famous.
6. Being anonymous online is fun because I don’t get in trouble for what I say. (New)
7. It’s easy to make other people angry when you are anonymous. (Rewritten)
8. When I am anonymous, I find it easier to trick and manipulate people. (New)
9. I like being anonymous because I can say whatever I want without consequences. (New)

**Factor 3: Anonymity Indifference (Not Included in Final Manuscript)**

Note, when developing and re-wording items between Study 1 and Study 2 we originally intended to include a third factor that we labelled anonymity indifference. This factor was subsequently removed from the manuscript due to its conceptual similarities with the two other factors in the scale. The below information is included for transparency.

From Study 1, the third factor *Anonymity Indifference* contained three items addressing an indifference to seeking anonymity. From Study 1 this factor was potentially underrepresented in the initial item pool and therefore six new items were written to better address the construct. Specifically, items were added to address people who believe they behave similarly in anonymous and identifiable scenarios, to address people who prefer identifiable environments, and finally to address people who see no benefit of the gratification’s anonymity affords. Finally, one item was rewritten for clarity.

***Initial Items from the First Exploratory Factor Analysis***

1. Being anonymous is no more fun than in real life.
2. I don’t express myself any better when I’m anonymous. (Rewritten)
3. When I’m anonymous online, I am no more honest than I am in the real world.

***Item Generation for the Second Exploratory Factor Analysis***

1. I don’t need anonymity to express myself any better. (Rewritten)
2. When I’m anonymous online, I am no more honest than I am in the real world.
3. Being anonymous online is no more fun than in real life.
4. I don’t really care if I’m anonymous online or not. (New)
5. My personality doesn’t change if I am anonymous online. (New)
6. I’d prefer to talk to someone in person than anonymously online. (New)
7. It’s easier to talk to people face to face than online. (New)
8. I have no need for anonymity when I am online. (New)
9. I don’t really think about how anonymous I am when I’m online. (New)

**Study 2: The Online Anonymity Questionnaire**

This questionnaire is focused on why people are sometimes anonymous on the Internet. Please respond to the statements on anonymity below by indicating how much you agree/disagree with each.

**Self-Expression**

1. I feel more comfortable disclosing information about my ideas, thoughts and feelings when I am anonymous.
2. Being anonymous allows me to share thoughts and feelings I otherwise wouldn’t share with people who know me.
3. Being anonymous allows me to experiment with new ideas.
4. I feel like I can be somebody else when I am anonymous.
5. I can present myself in a different way when I’m anonymous.
6. Being anonymous online allows me to escape or distracted myself from reality.
7. When I am anonymous online, I can talk to people who wouldn’t normally talk to me in the offline world.
8. Being anonymous allows me to join groups I wouldn’t normally join in the real world.
9. I can connect with people I normally wouldn’t when I’m anonymous.
10. I feel like I can express my true self when I am anonymous.

**Online Toxicity**

1. I am more likely to do things that are unlawful or illegal when I am anonymous.
2. When I’m anonymous I do things that are normally unacceptable in society.
3. Being anonymous online makes me feel lonelier.
4. I get satisfaction from aggravating people anonymously online.
5. I think being anonymous could allow me to be famous.
6. Being anonymous online is fun because I don’t get in trouble for what I say.
7. It’s easy to make other people angry when you are anonymous.
8. When I am anonymous, I find it easier to trick and manipulate people.
9. I like being anonymous because I can say whatever I want without consequences.

**Anonymity Indifference (Not included in Manuscript)**

1. I don’t need anonymity to express myself any better.
2. When I’m anonymous online, I am no more honest than I am in the real world.
3. Being anonymous online is no more fun than in real life.
4. I don’t really care if I’m anonymous online or not.
5. My personality doesn’t change if I am anonymous online.
6. I’d prefer to talk to someone in person than anonymously online.
7. It’s easier to talk to people face to face than online.
8. I have no need for anonymity when I am online.
9. I don’t really think about how anonymous I am when I’m online.

**Study 3: The Online Anonymity Questionnaire – Final Factor Structure**

This questionnaire is focused on why people are sometimes anonymous on the Internet. Please respond to the statements on anonymity below by indicating how much you agree/disagree with each.

1. I feel more comfortable disclosing information about my ideas, thoughts, and feelings when I am anonymous *online*.
2. Being anonymous *online* allows me to share thoughts and feelings I otherwise wouldn’t share with people who know me.
3. Being anonymous *online* allows me to experiment with new ideas.
4. I feel like I can be somebody else when I am anonymous *online*.
5. I can present myself in a different way when I’m anonymous *online*.
6. Being anonymous online allows me to escape or distract myself from reality.
7. When I am anonymous online, I can talk to people who wouldn’t normally talk to me in the offline world.
8. Being anonymous *online* allows me to join groups I wouldn’t normally join in the real world.
9. I can connect with people I normally wouldn’t when I’m anonymous *online*.
10. I feel like I can express my true self when I am anonymous *online*.
11. I am more likely to do things that are unlawful or illegal when I am anonymous *online*.
12. When I’m anonymous *online* I do things that are normally unacceptable in society.
13. I get satisfaction from aggravating people anonymously online.
14. Being anonymous online is fun because I don’t get in trouble for what I say.
15. When I am anonymous *online*, I find it easier to trick and manipulate people.
16. I like being anonymous *online* because I can say whatever I want without consequences.

**Scoring Procedure**

Anonymous Self-Expression: 10 Items

1, 2, 3, 4, 5, 6, 7, 8, 9, 10

Anonymous Toxicity: 6 Items

11, 12, 13, 14, 15, 16

Scale Ranges from 1 (strongly disagree) to 5 (strongly agree)

No reverse scoring on any items

*Note.* Italicized words were not tested in this manuscript. However, these words have been included to ensure that participants are aware that each item pertains to anonymity in online environments. An additional study included the italicized words and found similar results. See Study A of the supplemental materials. For clarity, researchers may choose to include the italicized words to in future studies.

**Items not Included in the Final Manuscript:**

1. I don’t need anonymity to express myself any better.
2. Being anonymous online is no more fun than in real life.
3. I don’t really care if I’m anonymous online or not.
4. My personality doesn’t change if I am anonymous online.
5. I’d prefer to talk to someone in person than anonymously online.
6. It’s easier to talk to people face to face than online.
7. I have no need for anonymity when I am online.
8. I don’t really think about how anonymous I am when I’m online.

Anonymity Indifference: 8 Items

17, 18, 19, 20, 21, 22, 23, 24

**Table B**

*Item Level Correlations of the Online Anonymity Questionnaire (Study 3)*

| Variable | SE1 | SE2 | SE3 | SE4 | SE5 | SE6 | SE7 | SE8 | SE9 | SE10 | TOX1 | TOX2 | TOX3 | TOX4 | TOX5 |
| --- | --- | --- | --- | --- | --- | --- | --- | --- | --- | --- | --- | --- | --- | --- | --- |
| SE1 |  |  |  |  |  |  |  |  |  |  |  |  |  |  |  |
| SE2 | .96** |  |  |  |  |  |  |  |  |  |  |  |  |  |  |
| SE3 | .94** | .94** |  |  |  |  |  |  |  |  |  |  |  |  |  |
| SE4 | .87** | .90** | .92** |  |  |  |  |  |  |  |  |  |  |  |  |
| SE5 | .91** | .92** | .95** | .94** |  |  |  |  |  |  |  |  |  |  |  |
| SE6 | .94** | .94** | .95** | .92** | .93** |  |  |  |  |  |  |  |  |  |  |
| SE7 | .91** | .92** | .91** | .85** | .90** | .92** |  |  |  |  |  |  |  |  |  |
| SE8 | .91** | .92** | .94** | .89** | .93** | .92** | .93** |  |  |  |  |  |  |  |  |
| SE9 | .93** | .92** | .92** | .87** | .91** | .93** | .95** | .94** |  |  |  |  |  |  |  |
| SE10 | .94** | .96** | .93** | .88** | .92** | .91** | .90** | .91** | .90** |  |  |  |  |  |  |
| TOX1 | .57** | .60** | .59** | .69** | .66** | .65** | .56** | .62** | .58** | .52** |  |  |  |  |  |
| TOX2 | .67** | .69** | .69** | .78** | .73** | .75** | .70** | .71** | .70** | .66** | .81** |  |  |  |  |
| TOX3 | .56** | .60** | .59** | .68** | .62** | .63** | .59** | .60** | .56** | .58** | .74** | .91** |  |  |  |
| TOX4 | .77** | .81** | .81** | .88** | .83** | .83** | .80** | .80** | .78** | .77** | .76** | .90** | .84** |  |  |
| TOX5 | .68** | .72** | .75** | .82** | .77** | .75** | .70** | .74** | .71** | .70** | .78** | .85** | .82** | .86** |  |
| TOX6 | .77** | .82** | .80** | .88** | .83** | .83** | .79** | .78** | .77** | .80** | .72** | .90** | .86** | .96** | .85** |

*Note.* SE = Anonymous Self-Expression, TOX = Toxic Anonymity.

**Table C**

*Descriptive Statistics of Online Behaviors and Factor Loadings (Study 3c)*

|  | **Mean** | **SD** | **Factor 1** | **Factor 2** |
| --- | --- | --- | --- | --- |
| Chatting | 2.20 | 0.93 |  | **.637** |
| Building relationships | 1.86 | 0.93 |  | **.819** |
| Debating | 1.96 | 0.93 | .336 | **.496** |
| Sharing secrets | 1.46 | 0.73 | .119 | **.408** |
| Ghosting | 1.82 | 0.90 |  | **.393** |
| Being antagonistic | 1.67 | 0.72 | **.767** |  |
| Catfishing | 1.41 | 0.69 | **.527** |  |
| Trolling | 1.44 | 0.78 | **.724** |  |
| Treating people badly | 1.46 | 0.67 | **.829** |  |
| Upsetting Other People | 1.62 | 0.79 | **.580** | .173 |

*Note. SD* = Standard Deviation. Factor 1 = Malign Behavior, Factor 2 = Benign Behavior

**Study 4: Daily Diary Study Procedure**

After providing consent, participants first completed an initial baseline survey, including questions relating to their age, gender, ethnicity, self-esteem, self-concept clarity, the Dark Triad, sadism, and the OAQ. Participants then completed a seven-day diary study. Each day, participants completed a short questionnaire asking them to report how much time they spent participating in *active* online behavior while identifiable and anonymous from the previous day (e.g., on Monday, participants reported their behavior for Sunday). Participants were also asked what behaviors they had engaged in the previous day, using the same behaviors from Study3c, with the addition of posting and the exclusion of lurking as it is a passive behavior.

The difference between identifiable and anonymous use was explained to participants as follows: “When using the Internet, sometimes we are identifiable and feel that other people can see who we are. At other times, however, we are anonymous and feel that other people do not know who we are”. Active use was then defined to participants as “engaging and interacting with the website (i.e., posting, commenting, replying, liking, communicating).” It was further noted that this definition did not include passive use (i.e., scrolling, lurking, watching videos).

For the daily questionnaires, participants were first asked if they had actively used social media or forum sites while *identifiable* in the past day. If they selected yes, they were subsequently asked how much time they had spent actively using social media or forum sites while identifiable in the past day. Previous research has suggested that people typically overestimate their time on social media, especially when responding to open-ended questions (Verbeij et al., 2021). As such, we used a seven-point ordinal scale, which is more precise for the first hour of use and broader for subsequent hours. This equates to the least error when collecting time spent using social media data via self-report (Ernala et al., 2020). This scale we used is as follows: 1 *= 1 to 10 minutes,* 2 *= 11 to 30 minutes,* 3 *= 31 to 59 minutes,* 4 *= one to two hours,* 5 *= two to three hours,* 6 *= three to five hours,* 7 *= more than five hours*. Next, participants were asked to indicate whether they had engaged in any of eleven online behaviors in the previous day, using the same behaviors from Study 3c, with the addition of posting and the exclusion of lurking as it is a passive behavior. Malign behavior included the following online behaviors: treating others badly, trolling, being antagonistic, catfishing, and upsetting other users. Benign behavior had the following online behaviors: building relationships, chatting, debating, sharing secrets, ghosting, and posting. After summing behaviors across the seven days, the event rate for some categories of behaviors was low. Accordingly, we scored these variables as 0 for no relevant behaviors and 1 for one or more relevant behaviors. Next, the same questions were again asked relating to participants' *anonymous* online behavior of the previous day.

Participants completed the same daily questionnaire for seven days, beginning on Monday and finishing on Sunday. Participants received the questionnaire at 9:00 a.m. via email, and the questionnaire was open for 8-hours. We recognize that there could be errors when recalling one’s online behavior for the previous day. However, an end-of-day questionnaire about one’s online behavior that day could miss relevant activities that happen after filling out the questionnaire. On Sunday, which was the final daily questionnaire, participants were debriefed and received their course credit.

**Test-Retest Study**

***Participants***

A power analysis indicated that a sample of 137 participants would provide 80% power to detect a medium effect size (*r* = .30). A sample of 189 participants (80 women; 102 men; 7 non-binary) aged between 18 and 76 (*M* = 32.87, *SD* = 12.52) was collected through Prolific. Participants were paid as completed the study in exchange for payment. Inclusion criteria were that only participants living in the United Kingdom, the USA, Canada, or Australia could complete the study. All participants’ primary language was English, and 74% of participants reported being White, 17% Asian, 4% African American, 2% Hispanic/Latino, and 3% Other. Of this sample, 169 participants (89%) completed a second questionnaire four weeks later. Participants were paid £2 for completing the first questionnaire and were subsequently paid an additional £2 for completing the second questionnaire four weeks later.

**Study A: Inclusion of Negatively Worded Items in the Online Anonymity Questionnaire**

In this study we made two changes to the wording of items in the online anonymity questionnaire (OAQ) and then reassessed the factor structure and model fit of the scale to see if it is consistent with the results seen in Study 3 of the manuscript. Some researchers argue that a combination of both positively and negatively worded items reduces the possibility of response bias and other method effects (Hinkin, 1995). To address the possibility of response bias we changed the wording of four items in the OAQ to become negatively worded. Further, to reduce ambiguity, improve content validity, and allow for the OAQ to be used in multi-scale surveys, we changed the wording of twelve items to explicitly mention the fact that the items were pertaining to online behaviour. The specific changes are outlined in the measures and procedures section below.

**Participants**

A sample of 322 Prolific workers (135 women; 179 men; 4 non-binary; 3 undisclosed) aged between 18 and 90 (*M* = 37.22, *SD* = 14.12) and living in the United Kingdom, the USA, Canada, or Australia participated in exchange for payment. All participants were fluent in English. The sample size was identical to the number of participants collected in Study 3 of the manuscript. Three participants were excluded for failing to pass an attention check (final *N* = 319). In the sample, 72% identified as White, 16% Asian, 4% African American, 2% Hispanic/Latino, and 6% Other.

**Measures and Procedure**

In Study A, participants completed the 16-item OAQ with a number of changes made to the wording of some items in the scale. The following changes were made. Two items were negatively worded for both the anonymous self-expression and anonymous toxicity subscales. For anonymous self-expression, these were: “Being anonymous online *does not* allow me to experiment with new ideas” and “I *cannot* present myself in a different way when I am anonymous online”. For anonymous toxicity, these were: “I *do not* get satisfaction from aggravating people anonymously online” and “When I am anonymous online, I *do not* find it easier to trick and manipulate people”. Additionally, twelve items in the OAQ were adjusted to explicitly mention that that the question is in relation to being anonymous online. See Table A to view changes to each item in the OAQ.

After providing informed consent, participants read that the purpose of the study was to investigate why people are sometimes anonymous on the internet. Participants first completed the 16-item OAQ before completing a series of demographic questions. The OAQ was rated on a 5-point scale (1 = *strongly disagree*, 5 = *strongly agree*). The order of the items was randomized for each participant.

**Statistical Approach**

Data analysis was conducted in R, primarily using the *psych* and *lavaan* packages (R Core Team, 2017; Revelle, 2020; Rosseel, 2012). We first conducted an exploratory factor analysis (EFA) to assess whether the item wording direction or item content influenced the scale’s factor structure. For the exploratory factor analysis, we used Promax rotation and principal axis factoring. The scale’s factor solution was chosen using parallel analysis. No items showed high intercorrelations (r > |.90|) or excessive skew (> |2|). We then conducted a confirmatory factor analysis (CFA) to assess model fit when including negatively worded items. We used the same model as used in Study 3 to compare both results. To determine acceptable model fit we used the following cut offs: CFI > 0.90, RMSEA < 0.07, and SRMR < 0.08 (Cheung & Rensvold, 2002).

**Results**

***Descriptive Statistics***

Item means and standard deviations for all positively worded items remained similar to earlier versions of the OAQ (see Table B). Further, the standard deviations for negatively worded items remained similar to versions of those items when they were positively worded.

***Exploratory Factor Analysis***

Parallel analysis indicated that there are three factors in the model. A subsequent EFA (Promax rotation, three-factors, principal axis factoring) showed that the negatively worded items for the anonymous self-expression subscale loaded onto their own factor (see Table A). A three-factor solution was returned, including 15-items and explaining 45% of the variance. Assumptions of sampling adequacy (Kaiser-Myer-Olkin = .89) and sphericity were met 𝜒2(120) = 1974.72, p < .001. The first factor (8-items, loadings = 0.62 – 0.83) included all positively worded items in the anonymous self-expression subscale. The second factor (5-items, loadings = 0.58 – 0.69) included four positively worded items, and one negatively worded item from the anonymous toxicity subscale. Finally, the third factor (2-items, loadings = 0.60 – 0.61) included two negatively worded items from the anonymous self-expression subscale. One item, “*When I am anonymous online, I do not find it easier to trick and manipulate people*” did not significantly load onto any factor.

**Table D**

*Factor Loadings from the Exploratory Factor Analysis in Study A*

| Item | Factor 1 | Factor 2 | Factor 3 |
| --- | --- | --- | --- |
| I feel more comfortable disclosing information about my ideas, thoughts, and feelings when I am anonymous ***online.*** | **.755** |  |  |
| Being anonymous ***online*** allows me to share thoughts and feelings I otherwise would not share with people who know me. | **.832** |  |  |
| Being anonymous ***online*** ***does not*** allow me to experiment with new ideas. (R) | .163 | -.113 | **.600** |
| I feel like I can be somebody else when I am anonymous ***online.*** | **.624** | .143 |  |
| I ***cannot*** present myself in a different way when I am anonymous ***online.*** (R) | .108 |  | **.609** |
| Being anonymous online allows me to escape or distract myself from reality. | **.653** |  |  |
| When I am anonymous online, I can talk to people who would not normally talk to me in the offline world. | **.729** |  |  |
| Being anonymous ***online*** allows me to join groups I would not normally join in the real world. | **.663** |  |  |
| I can connect with people I normally would not when I’m anonymous ***online.*** | **.751** |  |  |
| I feel like I can express my true self when I am anonymous ***online.*** | **.698** |  |  |
| I am more likely to do things that are unlawful or illegal when I am anonymous ***online.*** |  | **.610** | -.106 |
| When I am anonymous ***online,*** I do things that are normally unacceptable in society. | .160 | **.622** |  |
| I ***do not*** get satisfaction from aggravating people anonymously online. (R) | -.226 | **.583** | .156 |
| Being anonymous online is fun because I don’t get in trouble for what I say. | .245 | **.585** |  |
| When I am anonymous ***online***, I ***do not*** find it easier to trick and manipulate people. (R) | -.103 | .106 | .326 |
| I like being anonymous ***online*** because I can say whatever I want without consequences. | .175 | **.688** |  |

*Note.* Factor loadings reflect values from the pattern matrix. Bolded coefficients denote primary factor loadings. Factor loadings < .01 are removed. Items with an (R) symbol denote that the item is negatively worded. Italicized words indicate changes to the item from previous versions of the scale.

***Confirmatory Factor Analysis***

We aimed to fit the two-factor structure seen in Study 3 of the manuscript. The two-factor solution showed acceptable model fit 𝒳^2^(103) = 277.16, *p* < .001, CFI = 0.91, RMSEA = 0.07, SRMR = 0.06. However, one fit index showed larger than expected covariance not captured by the initial model structure. Modification indices suggested that one item pair (the negatively worded items in the self-expression subscale) showed larger than expected covariances. Parameter constraints were freed for this item pair. The modified three-factor structure demonstrated improved model fit, 𝒳^2^(100) = 234.44, *p* < .001, 𝒳^2^Δ (246) = 43.72, *p* < .001, CFI = 0.93, RMSEA = 0.06, SRMR = 0.06.

Loadings for the anonymous self-expression subscale ranged between .23 - .83, with both negatively worded items being below the threshold of .30 (.23 and .27, respectively). For anonymous toxicity factor loadings ranged between .12 and .81. One negatively worded item was below the threshold (loading = .12) whereas the other negatively worded item was within the accepted threshold (loading = .43). See Table B. Anonymous self-expression and toxicity anonymity were positively correlated (*r* = .46, p < .001). Internal reliability was acceptable for both factors (anonymous self-expression = .80, anonymous toxicity = .73).

**Table E**

*Comparison of Descriptive Statistics and Factor Loadings from Confirmatory Factor Analyses in Study 3 (Manuscript) and Study A (Negative Items)*

|  |  | **Study A (Negative Items)** | | | **Study 3 (Manuscript)** | | |
| --- | --- | --- | --- | --- | --- | --- | --- |
| Number | Items | Mean | SD | Loadings | Mean | SD | Loadings |
| SE1 | I feel more comfortable disclosing information about my ideas, thoughts, and feelings when I am anonymous ***online.*** | 3.50 | 1.18 | .74 | 3.37 | 1.30 | .73 |
| SE2 | Being anonymous ***online*** allows me to share thoughts and feelings I otherwise would not share with people who know me. | 3.44 | 1.21 | .83 | 3.23 | 1.31 | .75 |
| SE3 | Being anonymous ***online*** ***does not*** allow me to experiment with new ideas. (R) | 3.71 | 0.93 | .27 | 3.24 | 1.26 | .79 |
| SE4 | I feel like I can be somebody else when I am anonymous ***online.*** | 3.04 | 1.65 | .71 | 2.88 | 1.27 | .70 |
| SE5 | I ***cannot*** present myself in a different way when I am anonymous ***online.*** (R) | 3.41 | 1.15 | .23 | 3.28 | 1.22 | .76 |
| SE6 | Being anonymous online allows me to escape or distract myself from reality. | 3.20 | 1.19 | .65 | 3.02 | 1.35 | .74 |
| SE7 | When I am anonymous online, I can talk to people who would not normally talk to me in the offline world. | 3.34 | 1.38 | .71 | 3.27 | 1.28 | .66 |
| SE8 | Being anonymous ***online*** allows me to join groups I would not normally join in the real world. | 3.13 | 1.29 | .70 | 3.08 | 1.31 | .71 |
| SE9 | I can connect with people I normally would not when I’m anonymous ***online.*** | 3.20 | 1.23 | .73 | 3.37 | 1.23 | .68 |
| SE10 | I feel like I can express my true self when I am anonymous ***online.*** | 3.22 | 1.18 | .74 | 3.09 | 1.24 | .71 |
| TOX1 | I am more likely to do things that are unlawful or illegal when I am anonymous ***online.*** | 1.85 | 1.08 | .53 | 1.96 | 1.18 | .52 |
| TOX2 | When I am anonymous ***online,*** I do things that are normally unacceptable in society. | 1.97 | 1.16 | .66 | 1.91 | 1.13 | .71 |
| TOX3 | I ***do not*** get satisfaction from aggravating people anonymously online. (R) | 1.96 | 1.15 | .43 | 1.69 | 1.05 | .63 |
| TOX4 | Being anonymous online is fun because I don’t get in trouble for what I say. | 2.53 | 1.20 | .75 | 2.40 | 1.22 | .76 |
| TOX5 | When I am anonymous ***online***, I ***do not*** find it easier to trick and manipulate people. (R) | 2.94 | 1.09 | .12 | 2.30 | 1.19 | .64 |
| TOX6 | I like being anonymous ***online*** because I can say whatever I want without consequences. | 2.38 | 1.23 | .81 | 2.39 | 1.26 | .75 |

*Note.* SD = Standard Deviation, SE = Anonymous Self-Expression, TOX = Toxic Anonymity. Items with an (R) symbol denote that the item is negatively worded in Study A. Italicized words indicate changes to the item from Study 3 to Study A. Means for negatively worded items in Study A have been reversed.

**Discussion**

In this study we aimed to assess the factor structure and model fit of the OAQ when negatively worded items were included, and all items explicitly mentioned that the question pertained to online behaviour. Results from the CFA indicated that the inclusion of negatively worded items reduced model fit, and that factor loadings for three of the four negatively worded items were below the minimum threshold of 0.40, indicating that those items should be removed from the model. Further, the EFA further indicated that the negatively worded that were supposed to load onto the anonymous self-expression subscale, instead loaded onto their own factor. Additionally, one negatively worded item that was supposed to load onto the anonymous toxicity subscale did not significantly load onto any factor.

Some researchers have noted that the use of negatively oriented items, while well intentioned, can result in poor measurement and unintended psychometric issues (Dalal & Carter, 2014; Podsakoff et al., 2003). Using positively and negatively worded items can lead to separate method effects, more measurement error, less reliability, and does not necessarily fix issues of attentiveness and impression management (Cole et al., 2019; Dalal & Carter, 2014; Dueber et al., 2022; Lindwall et al., 2012; Sonderen et al., 2013). Further, a key assumption of a mixed-item approach is that the positively and negatively worded items measure the same underlying construct (Marsh, 1996). However, the measurement error caused by negatively oriented items often generates an independent factor within an exploratory factor analysis. This factor represents an artefact of the language of the item rather than its underlying construct (Dueber et al., 2022). In our case, it is possible that the meaning of some of items that we negatively worded changed, leading to some negatively worded items loading onto their own factor.

The results from exploratory factor analysis revealed that all positively phrased items of the OAQ remained in the same factor as seen in Study 3 and showed similar means and standard deviations. Additionally, these factors remained internally consistent (anonymous self-expression α = .90, anonymous toxicity α = .78) when only positively worded items were included in the model. This indicates that not explicitly mentioning the online nature of the scale within each item is unlikely to have influenced the results of the manuscript. Potentially, this is because participants are informed that the scale pertains to online anonymity before they begin the questionnaire. Nonetheless, we believe that having all items specifically pertain to online anonymity is important. This is because doing so improves the content validity of the scale and allows the scale to be effectively used in multi-scale surveys. As such, we suggest that all future studies who choose to use the OAQ should use the updated version.

**References**

Cole, K. L., Turner, R. C., & Gitchel, W. D. (2019). A study of polytomous IRT methods and item wording directionality effects on perceived stress items. *Personality and Individual Differences*, *147*, 63–72. https://doi.org/10.1016/j.paid.2019.03.046

Dalal, D. K., & Carter, N. T. (2014). Negatively worded items negatively impact survey research. In *More statistical and methodological myths and urban legends* (pp. 112–132). Routledge.

Dueber, D. M., Toland, M. D., Lingat, J. E., Love, A. M. A., Qiu, C., Wu, R., & Brown, A. V. (2022). To Reverse Item Orientation or Not to Reverse Item Orientation, That Is the Question. *Assessment*, *29*(7), 1422–1440. https://doi.org/10.1177/10731911211017635

Hinkin, T. R. (1995). A review of scale development practices in the study of organizations. *Journal of Management*, *21*(5), 967–988. https://doi.org/10.1016/0149-2063(95)90050-0

Kam, C. C. S., & Meyer, J. P. (2015). How Careless Responding and Acquiescence Response Bias Can Influence Construct Dimensionality: The Case of Job Satisfaction. *Organizational Research Methods*, *18*(3), 512–541. https://doi.org/10.1177/1094428115571894

Lindwall, M., Barkoukis, V., Grano, C., Lucidi, F., Raudsepp, L., Liukkonen, J., & Thøgersen-Ntoumani, C. (2012). Method Effects: The Problem With Negatively Versus Positively Keyed Items. *Journal of Personality Assessment*, *94*(2), 196–204. https://doi.org/10.1080/00223891.2011.645936

Marsh, H. W. (1996). Positive and negative global self-esteem: A substantively meaningful distinction or artifactors? *Journal of Personality and Social Psychology*, *70*, 810–819. https://doi.org/10.1037/0022-3514.70.4.810

Podsakoff, P. M., MacKenzie, S. B., Lee, J.-Y., & Podsakoff, N. P. (2003). Common method biases in behavioral research: A critical review of the literature and recommended remedies. *Journal of Applied Psychology*, *88*, 879–903. https://doi.org/10.1037/0021-9010.88.5.879

R Core Team. (2017). *R: A Language and Environment for Statistical Computing*. https://www.r-project.org/

Revelle, W. (2020). *psych: Procedures for Psychological, Psychometric, and Personality Research*. https://cran.r-project.org/web/packages/psych/citation.html

Rosseel, Y. (2012). lavaan: An R Package for Structural Equation Modeling. *Journal of Statistical Software*, *48*(2), 1–16. https://doi.org/10.18637/jss.v048.i02

Sonderen, E. van, Sanderman, R., & Coyne, J. C. (2013). Ineffectiveness of Reverse Wording of Questionnaire Items: Let’s Learn from Cows in the Rain. *PLoS ONE*, *8*(7), e68967. https://doi.org/10.1371/journal.pone.0068967

Zhang, X., Noor, R., & Savalei, V. (2016). Examining the Effect of Reverse Worded Items on the Factor Structure of the Need for Cognition Scale. *PLoS ONE*, *11*(6), e0157795. https://doi.org/10.1371/journal.pone.0157795
